# A predatory myxobacterium controls cucumber Fusarium wilt by regulating the soil microbial community

**DOI:** 10.1186/s40168-020-00824-x

**Published:** 2020-04-06

**Authors:** Xianfeng Ye, Zhoukun Li, Xue Luo, Wenhui Wang, Yongkai Li, Rui Li, Bo Zhang, Yan Qiao, Jie Zhou, Jiaqin Fan, Hui Wang, Yan Huang, Hui Cao, Zhongli Cui, Ruifu Zhang

**Affiliations:** 1 Key Laboratory of Agricultural Environmental Microbiology, Ministry of Agriculture and Rural Affairs, College of Life Science of Nanjing Agricultural University, Nanjing, 210095 People’s Republic of China; 2 Key Laboratory of Soil Environment and Pollution Remediation, Institute of Soil Science, Chinese Academy of Sciences, Nanjing, 210008 People’s Republic of China; 3 Key Laboratory of Monitoring and Management of Plant Diseases and Insects, Ministry of Agriculture and Rural Affairs, College of Plant Protection, Nanjing Agricultural University, Nanjing, 210095 People’s Republic of China; 4 Key Laboratory of plant immunity, Nanjing Agricultural University, Nanjing, 210095 People’s Republic of China; 5 Key Laboratory of Microbial Resources Collection and Preservation, Ministry of Agriculture, Institute of Agricultural Resources and Regional Planning, Chinese Academy of Agricultural Sciences, Beijing, 100081 People’s Republic of China

**Keywords:** Myxobacteria, Micropredator, *Corallococcus* sp. EGB, *Fusarium oxysporum* f. sp. *cucumerinum*, Soil microbiome, Root exudates, Cooccurrence network

## Abstract

**Background:**

Myxobacteria are micropredators in the soil ecosystem with the capacity to move and feed cooperatively. Some myxobacterial strains have been used to control soil-borne fungal phytopathogens. However, interactions among myxobacteria, plant pathogens, and the soil microbiome are largely unexplored. In this study, we aimed to investigate the behaviors of the myxobacterium *Corallococcus* sp. strain EGB in the soil and its effect on the soil microbiome after inoculation for controlling cucumber Fusarium wilt caused by *Fusarium oxysporum* f. sp. *cucumerinum* (FOC).

**Results:**

A greenhouse and a 2-year field experiment demonstrated that the solid-state fermented strain EGB significantly reduced the cucumber Fusarium wilt by 79.6% (greenhouse), 66.0% (2015, field), and 53.9% (2016, field). Strain EGB adapted to the soil environment well and decreased the abundance of soil-borne FOC efficiently. Spatiotemporal analysis of the soil microbial community showed that strain EGB migrated towards the roots and root exudates of the cucumber plants via chemotaxis. Cooccurrence network analysis of the soil microbiome indicated a decreased modularity and community number but an increased connection number per node after the application of strain EGB. Several predatory bacteria, such as *Lysobacter*, *Microvirga*, and *Cupriavidus*, appearing as hubs or indicators, showed intensive connections with other bacteria.

**Conclusion:**

The predatory myxobacterium *Corallococcus* sp. strain EGB controlled cucumber Fusarium wilt by migrating to the plant root and regulating the soil microbial community. This strain has the potential to be developed as a novel biological control agent of soil-borne Fusarium wilt.

Video abstract.

## Background

*Fusarium oxysporum* Schlecht. is a ubiquitous soil-borne phytopathogen that can cause vascular wilt in many widely cultivated crops [1]. Controlling vascular wilt pathogens is difficult due to the lack of efficient curative treatments for infected plants and the pathogens’ ability to survive for a long time in soil without a host plant [2]. Interest in the biocontrol of Fusarium wilts using antagonistic microorganisms has recently been renewed due to the absence of effective chemical control methods [3]. Numerous biological control agents (BCAs) have been studied, but only a limited number of strains from the genera *Bacillus*, *Pseudomonas*, *Streptomyces*, *Trichoderma*, *Coniothyrium*, and *Gliocladium* and nonpathogenic *Fusarium* have been commercially developed [4].

However, the use of registered BCAs is limited because of their poor efficacy and unstable performance [3]. Once applied to the soil, BCAs interact with the host plants, native soil microbes, target soil-borne pathogens, and the edaphic environment [5]. Understanding these ecological interactions is critical for the commercial development of BCAs [6]. In *Trichoderma hamatum* strain GD12, knockout of the *N*-acetyl-b-d-glucosaminidase gene reduced its competitive saprotrophic fitness and impaired its biocontrol ability [7]. Furthermore, plant microbiomes are crucial for plant health [8]. Biocontrol of plant soil-borne pathogens can also be achieved through the regulation of soil microbial communities [9, 10]. It has been reported that the reduction in soil microbial diversity was responsible for the burst of soil-borne plant diseases [11]. There has been an increased interest in the microbial communities of disease-suppressive soils, and these suppressive effects have been attributed to the enrichment of specific groups of soil microbes [12–15]. The fact that a community composed of non-antagonistic bacteria from multiple parallel mineralization systems could also suppress Fusarium wilt disease indicates the importance of the microbial community structure to the biocontrol capacity of BCAs [16].

Successful biocontrol depends not only on plant-microbe interactions but also on the ecological fitness of the BCAs [3, 6]. Generally, suppression of plant pathogens by BCAs is usually attributed to their competitive colonization of plant roots, secretion of antimicrobial compounds [17], and induction of systemic resistance responses in the plant hosts [18]. Mining of microbial resources adapted to complicated soil environments and equipped with novel biocontrol mechanisms is necessary for successful biocontrol of Fusarium wilt.

Myxobacteria are Gram-negative soil bacteria that display complex multicellular morphogenesis and social behaviors over their life cycles [19, 20]. Most myxobacteria can prey on various microorganisms, including bacteria and fungi [21–23]. Predatory bacteria have been proposed as potential biological agents and have shown effective biocontrol activity against various plant soil-borne pathogens [24–27]. Their abilities to slide on solid surfaces, significant capacity to produce antibiotics, formation of myxospores, and predatory behaviors make myxobacteria excellent candidates for BCAs.

Our previous work showed that the myxobacterium *Corallococcus* sp. EGB could control FOC by predation [25]. We hypothesize that predatory myxobacteria have advantages over other BCAs due to their ability to feed on pathogens and to survive in soils. The purposes of this study were (1) to investigate the biocontrol of Fusarium wilt and the ecological behaviors of strain EGB in the cucumber-*Fusarium* system and (2) to evaluate the effects of strain EGB on the soil microbial community. Our results revealed that myxobacterial predation of FOC and the alteration of the rhizosphere microbial community contributed to the efficient suppression of cucumber Fusarium wilt disease.

## Results

### Solid-state fermented strain EGB exhibited efficient biocontrol of cucumber Fusarium wilt in pot- and field-scale experiments

Previous results showed that liquid culture of strain EGB exhibited excellent biocontrol of cucumber Fusarium wilt in pot experiments [25]. Given that myxobacteria grown in liquid culture are prone to agglomerate and autolyze, they are not suitable for agricultural applications. Thus, we developed a solid-state fermentation process for the production of strain EGB. Strain EGB formed myxospores with solid-state fermentation and survived for more than 1 year in solid culture (Additional file 1: Table S1). To test the biocontrol efficiency of strain EGB solid culture, a pot experiment was set as follows: no treatment (no FOC or strain EGB solid culture, NT), FOC only (FOC), both FOC and EGB solid culture (EGBFOC), and strain EGB solid culture only (EGB). The cucumber Fusarium wilt disease incidence and FOC/EGB abundances under all treatments were investigated after 15 and 27 days, respectively. The strain EGB solid culture significantly reduced the incidence of cucumber Fusarium wilt in pot experiments with relative control efficiencies of 67.4% and 79.6% on days 15 and 27, respectively (Table 1). The abundance of strain EGB remained stable in the soils of EGB and EGBFOC treatments after 27 days, while the FOC decreased by more than one order of magnitude as revealed by qPCR (Table 1). Furthermore, the induction of the *Corallococcus* spp. was observed in the FOC treatment.

**Table 1:** Effects of strain EGB solid culture application on the disease incidences of cucumber Fusarium wilt, and quantities of strains FOC and EGB in soil surrounding cucumber roots

Treatments	15^th^ day		27^th^ day		*Fusarium oxysporum* f. sp. *cucumerinum* (FOC)		*Corallococcus* sp. EGB	
	Disease incidence (%)	Biocontrol efficiency (%)	Disease incidence (%)	Biocontrol efficiency (%)	Ct value	Log_10_ Copies g^−1^ soil	Ct value	Log_10_ Copies g^−1^ soil
NT	^−^	^−^	^−^	^−^	33.1 ± 0.34^a^	^−^	32.9 ± 0.06^a^	^−^
EGB	^−^	^−^	^−^	^−^	31.7 ± 0.54^b^	^−^	24.3 ± 0.70^d^	6.09 ± 0.18^a^
EGBFOC	15.5 ± 2.1^b^	67.4	14.3 ± 0.0^b^	79.6	30.2 ± 0.41^c^	4.53 ± 0.12^b^	26.3 ± 0.45^c^	5.56 ± 0.12^b^
FOC	47.6 ± 5.5^a^	^−^	70.2 ± 4.1^a^	^−^	25.3 ± 0.12^d^	5.91 ± 0.03^a^	30.6 ± 0.79^b^	4.42 ± 0.21^c^

Note: In greenhouse experiment, each treatment had three repeats and the data represent the means and standard deviations (mean ± SD) from three replications. In the same column, values designated with the same letters were not significantly different (*p* ≤ 0.05) according to Duncan’s test. NT, no FOC or strain EGB solid culture; EGB, strain EGB solid culture only; EGBFOC, both FOC and EGB solid culture; FOC, FOC only

**Fig. 1:**
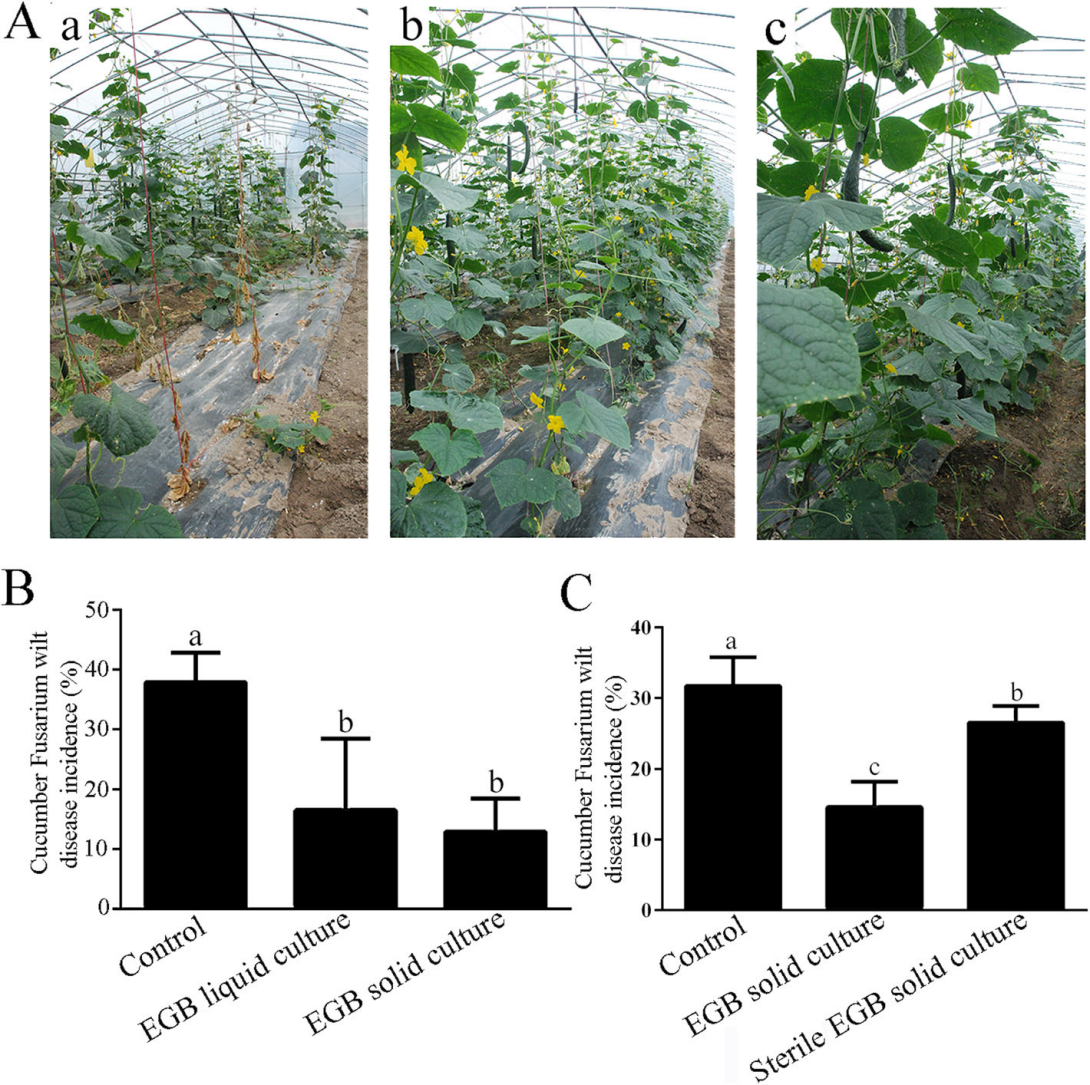
Two-year field performance of strain EGB in the control of cucumber Fusarium wilt. **A** Growth of cucumbers one-month post application of strain EGB in 2015; a Control without any treatment, b EGB liquid culture, and c EGB solid culture. **B, C** Statistical analysis of wilt disease incidences in 2015 (B) and 2016 (C). Two hundred cucumbers in each treatment were randomly divided into four blocks, and bars indicate the standard deviations of the averages from four replicates. Values designated with the same letters were not significantly different (*p* ≤ 0.05) according to Duncan’s test

A 2-year field experiment was carried out to test the biocontrol potential of strain EGB. In 2015, both liquid and solid cultures of strain EGB were employed, and the average disease incidences in EGB liquid culture and solid culture amended plots were 16.5% and 12.9% (Fig. 1A and B), corresponding to wilt disease reduction of 56.5% and 66.0%, respectively. The biocontrol efficiency was not significantly different between the EGB liquid culture and solid culture treatments, indicating that strain EGB solid culture could be developed as a myxobacterial BCA for Fusarium wilt.

Strain EGB solid culture continued to be applied in 2016 to validate the results of the previous year’s field experiment, with a sterilized solid culture added as a control to evaluate the contribution of the fermentation substances. The disease incidences of plots treated with EGB solid culture and sterilized solid culture were 14.6% and 26.5% (Fig. 1C), and the corresponding biocontrol efficiencies were 53.9% and 16.4%, respectively. The 2-year field experiment demonstrated that strain EGB exhibited efficient biocontrol of cucumber Fusarium wilt in the field.

### Strain EGB migrated towards cucumber roots in the soil

**Fig. 2:**
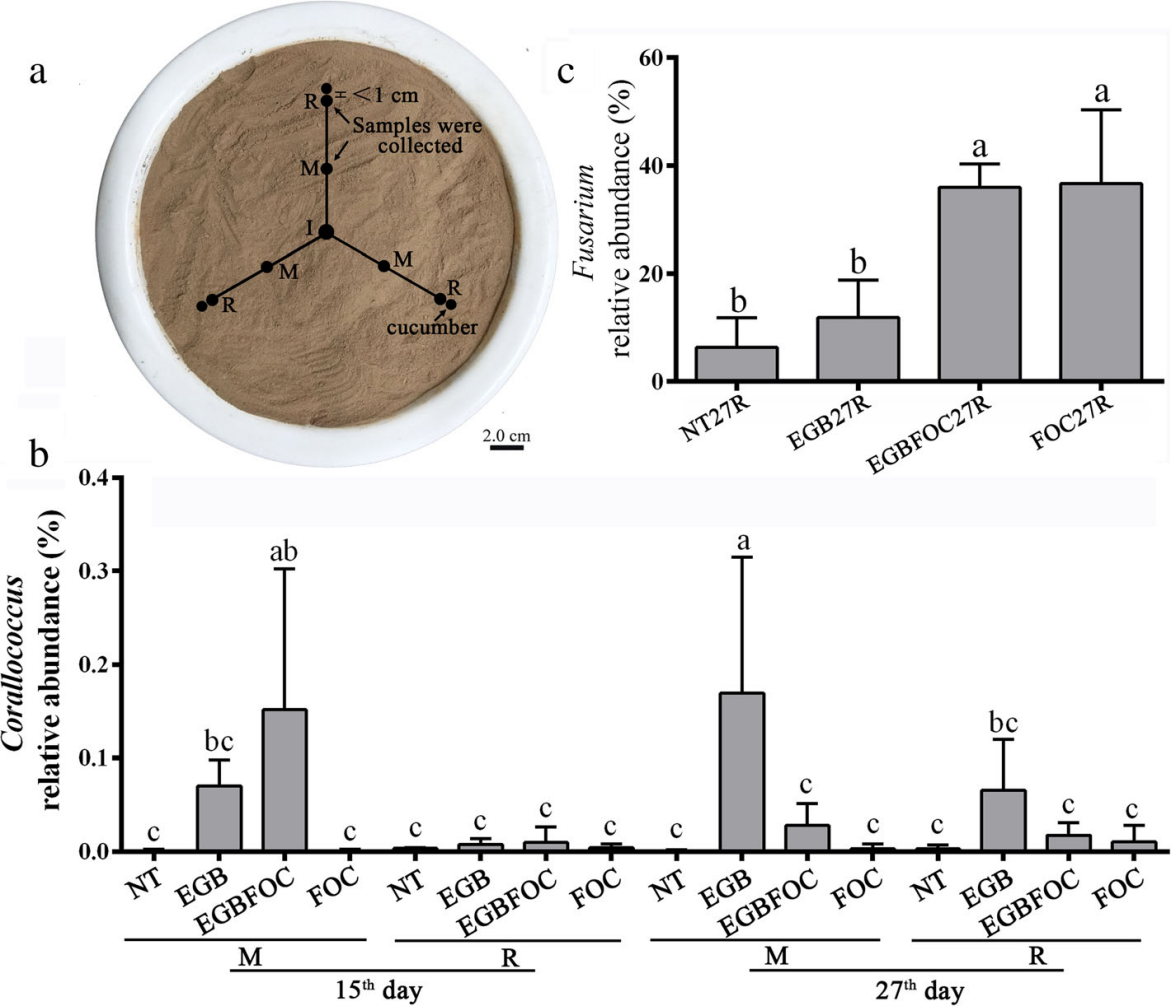
Sampling strategy (**a**) and relative spatiotemporal abundances of *Corallococcus* (**b**) and *Fusarium* (**c**). I, Strain EGB inoculation site; R, the sampling site surrounding the roots; M, a site between the cucumber root and inoculation site; NT, no FOC or strain EGB solid culture; EGB, strain EGB solid culture only; EGBFOC, both FOC and EGB solid culture; FOC, FOC only. Each sample had 3 repeats, and the bars represent the standard deviations of averages from three replicates. Values designated with the same letters were not significantly different (*p* ≤ 0.05) according to Duncan’s test

To elucidate the Fusarium wilt disease suppressive mechanism of strain EGB, the ecological behavior of strain EGB in soil was investigated in the above mentioned pot experiments (NT, EGB, EGBFOC, FOC). Strain EGB solid culture was inoculated into the center of a pot (I, Fig. 2a). Soil samples were collected from the R and M sites on the 15^th^ and 27^th^ day, respectively. The spatiotemporal distribution of *Corallococcus* was analyzed by 16S rRNA gene PCR amplicon high-throughput sequencing. The results showed that the relative abundances of *Corallococcus* in the control (NT) and FOC treatments remained at low levels during the entire experimental period (Fig. 2b). Nevertheless, the relative abundance of *Corallococcus* in treatments with strain EGB application (EGB and EGBFOC) showed regular variation depending on sampling site and time. The relative abundances of *Corallococcus* at the M sites of these two treatments with strain EGB applied were higher than those of the NT and FOC treatments on the 15^th^ day and were maintained at a relatively high level on the 27^th^ day. Moreover, the relative abundances of *Corallococcus* at the R sites showed an increasing trend on the 27^th^ day (Fig. 2b). In particular, the *Corallococcus* relative abundances at the M sites of the EGB treatment on the 27^th^ day or the EGBFOC treatment on the 15th day increased significantly (*p* < 0.05). However, the relative abundances of *Corallococcus* in the soils surrounding the roots were not significantly different on the 15^th^ day (NT15R, EGB15R, EGBFOC15R, and FOC15R). The successive changes in the relative abundance between the M and R sites in treatment of EGB and EGBFOC indicated that strain EGB could migrate from the inoculation site to the roots. The relative abundance of *Fusarium* was also investigated. In contrast to the significant decrease in FOC abundance (Table 1), the relative abundance of other members of the *Fusarium* genera in the soil was obviously increased after FOC inoculation (Fig. 2c). Enrichment of *Corallococcus*, which increased from 0.00077 to 0.0103%, was also observed in the FOC treatment (FOC27R), as verified by qPCR analysis (Table 1).

### Chemotaxis towards root exudates and cucumber root colonization by strain EGB

**Fig. 3:**
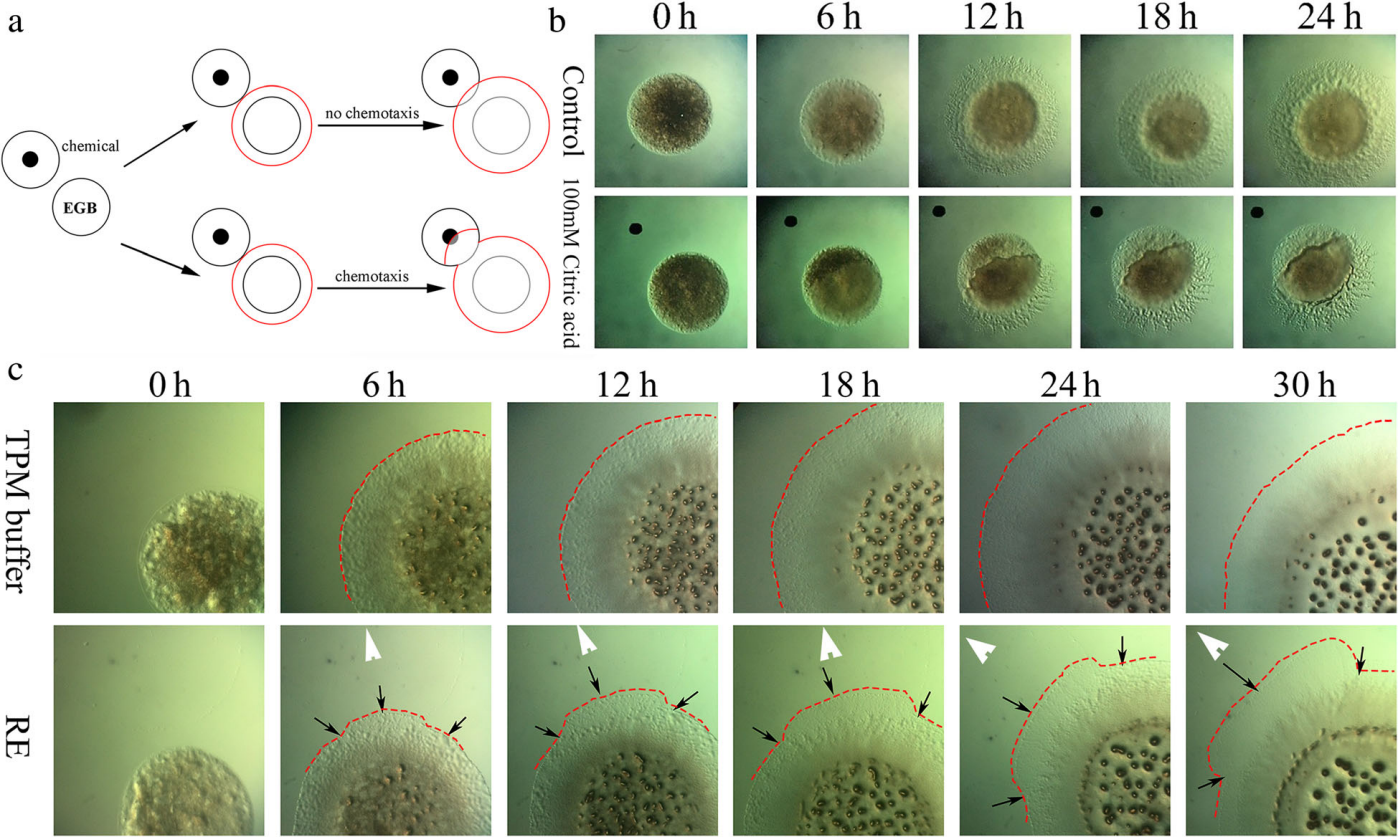
Chemotaxis of strain EGB towards cucumber root exudates on TPM plates. **a** Diagram of the chemotactic response of EGB to the chemicals. **b** Feasibility test of the chemotaxis assay with citrate acid. **c** Chemotaxis of strain EGB towards cucumber root exudate. RE, root exudates of cucumber; black arrow, sites where asymmetric swarming occurred; white arrow, sites where the chemicals were dropped; red dotted line, edge of the swarming colonies of strain EGB. The experiment was repeated four times, with three replicates each time

Root exudates play key roles in plant-microbe interactions and root colonization by rhizobacteria [28]. Based on the fact that strain EGB could migrate towards cucumber roots in soil, we deduced that root exudates play a role in the recruitment of strain EGB to the rhizosphere. Considering the slow movement of strain EGB on soft agar surfaces, we tested its chemotactic responses on a semisolid TPM plate as described in the materials and methods. Cells of strain EGB were clearly repelled by citric acid (0.1 M, pH 2) on semisolid TPM plates (Fig. 3b), indicating the feasibility of the plate assay for myxobacterial chemotaxis. To test strain EGB’s chemotaxis towards root exudates, 6-μl sterile cucumber root exudates were dropped on a semisolid TPM plate 2 mm away from the strain EGB colony (Fig. 3a). The chemotactic response of strain EGB towards the root exudates was observed under a light microscope (Fig. 3c). The results showed that EGB colonies grew towards the cucumber root exudates asymmetrically and finally covered the root exudate area after 48 h of incubation. In contrast, EGB colonies grew symmetrically outwards on the control TPM plates.

To identify which root secreted chemoattractants recruit EGB, 13 previously identified compounds in the root exudates were tested [29, 30]. Strain EGB showed chemotactic responses to 6 of them (Table 2). Interestingly, although sugars were not effective substrates for the growth of strain EGB (Additional file 2: Fig. S1), they were the most effective chemoattractants. Strain EGB was strongly attracted by maltose and maltitol at a concentration of 10 μM. Moderate to high concentrations of glucose and arabitol were weak chemoattractants of strain EGB. Among the tested amino acids, strain EGB showed weak chemotaxis towards relatively high concentrations of alanine and tryptophan. However, no chemotactic response was observed to any of the tested organic acids.

**Table 2:** The chemotactic response of strain EGB to 13 compounds in cucumber root exudates

Chemicals	Concentration							
	10 μM	30 μM	50 μM	80 μM	100 μM	1 mM	10 mM	100 mM
Citrate	^−^	^−^	^−^	^−^	^−^	^−^	^−^	^−^
Malate	^−^	^−^	^−^	^−^	^−^	^−^	^−^	^−^
Succinate	^−^	^−^	^−^	^−^	^−^	^−^	^−^	^−^
Fumarate	^−^	^−^	^−^	^−^	^−^	^−^	^−^	^−^
Alanine	^−^	^−^	^−^	^−^	**+**	**+**	**+**	^−^
Aspartic acid	^−^	^−^	^−^	^−^	^−^	^−^	^−^	^−^
Tryptophan	^−^	^−^	^−^	^−^	^−^	^−^	**+**	**+**
Valine	^−^	^−^	^−^	^−^	^−^	^−^	^−^	^−^
Sucrose	^−^	^−^	^−^	^−^	^−^	^−^	^−^	^−^
Maltose	**++**	**++**	**++**	**++**	**++**	**+**	**+**	^−^
Glucose	^−^	^−^	^−^	^−^	**+**	**+**	^−^	^−^
Maltitol	**++**	**++**	**++**	**++**	**+**	**+**	^−^	^−^
Arabitol	^−^	^−^	^−^	^−^	**+**	**+**	**+**	**+**

Note: “+”, positive chemotactic response, multiple + represent the strength of chemotactic response; “^−^”, no chemotactic response. The experiment was repeated three times with three sample replicates

**Fig. 4:**
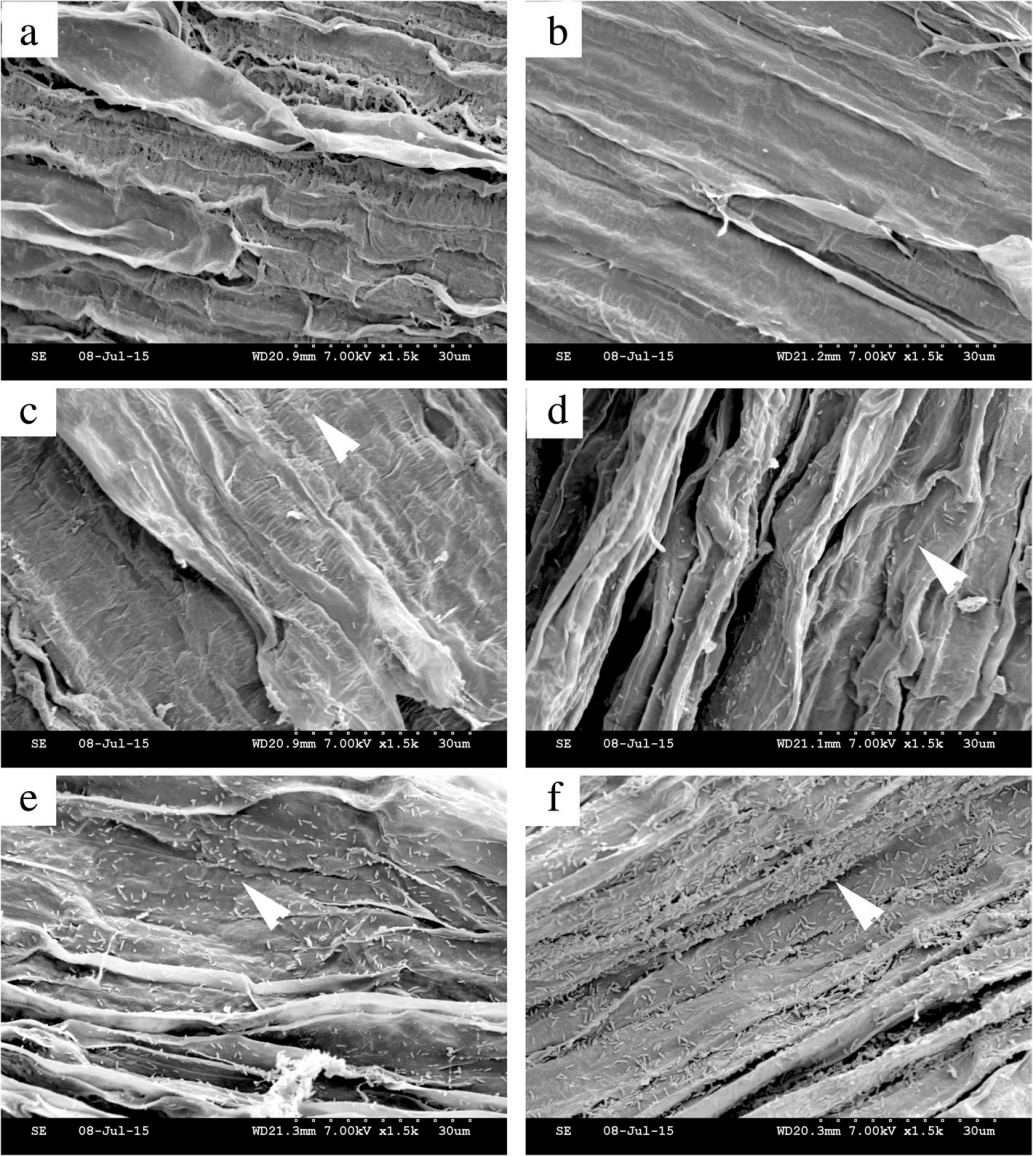
Colonization of cucumber roots by strain EGB. **a**, **c**, and **e** Meristematic zones of cucumber roots. **b**, **d**, and **f** Elongation zones of cucumber roots. **a**, **b** Blank TPM buffer control. **c**, **d***E. coli* DH10B negative control. **e**, **f** EGB treatment. Arrows, cells of *E. coli* DH10B or strain EGB

Root colonization is important for biocontrol agents to function. Strain EGB was chemoattracted by root exudates and moved towards cucumber roots. We then investigated the ability of strain EGB to colonize the cucumber roots under sterile conditions. *Escherichia coli* DH10B was included as a negative control due to its poor colonization of roots [31]. The cucumber seedlings were incubated in EGB-suspended TPM buffer for 24 h and then observed by SEM. The results showed that the strain EGB cells evidently colonized the elongation zone of the cucumber roots (Fig. 4f), and the meristematic zone was also observed to be colonized by EGB (Fig. 4e). However, the elongation zone was preferred by strain EGB, as revealed by the cell densities observed in the colonization of different root zones. The densities of *E. coli* DH10B cells in the elongation zone and meristematic zone were significantly lower than those of strain EGB (Fig. 4c, d). In contrast, cells of strain EGB were evenly distributed on the cucumber rhizoplane and formed a thin layer of biofilm (Fig. 4f).

### The application of strain EGB solid culture changed the soil microbial diversity

**Fig. 5:**
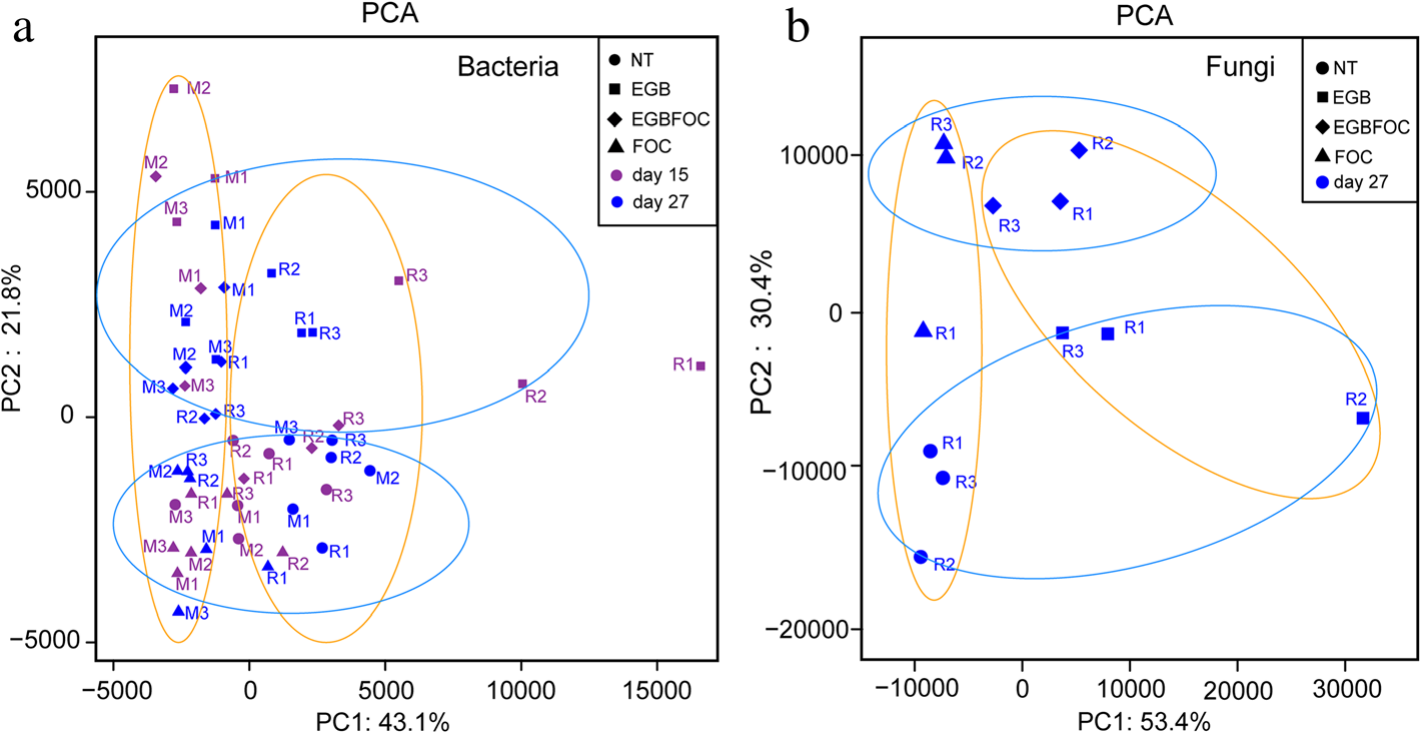
Principal component analysis of the soil bacterial (**a**) and fungal (**b**) communities. NT, no FOC or strain EGB solid culture; EGB, strain EGB solid culture only; EGBFOC, both FOC and EGB solid culture; FOC, FOC only. R, the sampling site surrounding the roots; M, the site between the inoculation site and the cucumber roots; 15, soils sampled on the 15th day; 27, soils sampled on the 27th day

Strain EGB has the ability to prey on various bacteria and fungi (Additional file 3: Fig. S2). We hypothesized that once strain EGB was applied to the soil, it would prey on soil bacteria and fungi, then alter the soil microbiome. To confirm this hypothesis, the soil microbial community diversity and composition of the pot experiment were analyzed by high-throughput sequencing (Additional file 4: Table S2 and Additional file 5: Fig. S3). The results showed that alpha diversity indices, including Chao, ACE, and Shannon, were decreased significantly (*p* < 0.05) on the 15th day, then recovered and stabilized on the 27th day in the EGB-amended treatments (EGB, EGBFOC) (Additional file 6: Fig. S4B and S4C). Principal component analysis (PCA) of the bacterial community showed that PC1 and PC2 explained 43.1% and 21.8% of the total variation (Fig. 5a). The structure of the bacterial communities in bulk (M) and the root surrounding soils (R) were obviously separated on the PC1-axis in the PCA, indicating the effect of the plants on the assembly of the soil microbial community. The EGB and EGBFOC treatments were clearly separated from the NT and FOC treatments on the PC2-axis, suggesting the role of strain EGB as a driver of bacterial community development (Fig. 5a).

The fungal diversity in cucumber root surrounding soils was also analyzed on 27^th^ day (Additional file 7: Table S3 and Additional file 8: Fig. S5). The alpha diversity index was not significantly different among these treatments (Additional file 9: Fig. S6). However, the PCA of the fungal community showed that PC1 and PC2 explained 53.4% and 30.4% of the total variation, respectively (Fig. 5b). All these samples could be separated on the PC1-axis with the application of the strain EGB solid culture or separated on the PC2-axis with the FOC. Hence, considering the community dynamics after the application of the strain EGB solid culture, we deduced that predation by strain EGB may be an important driving force of microbial community alteration.

### The application of strain EGB solid culture changed the microbial cooccurrence networks

**Fig. 6:**
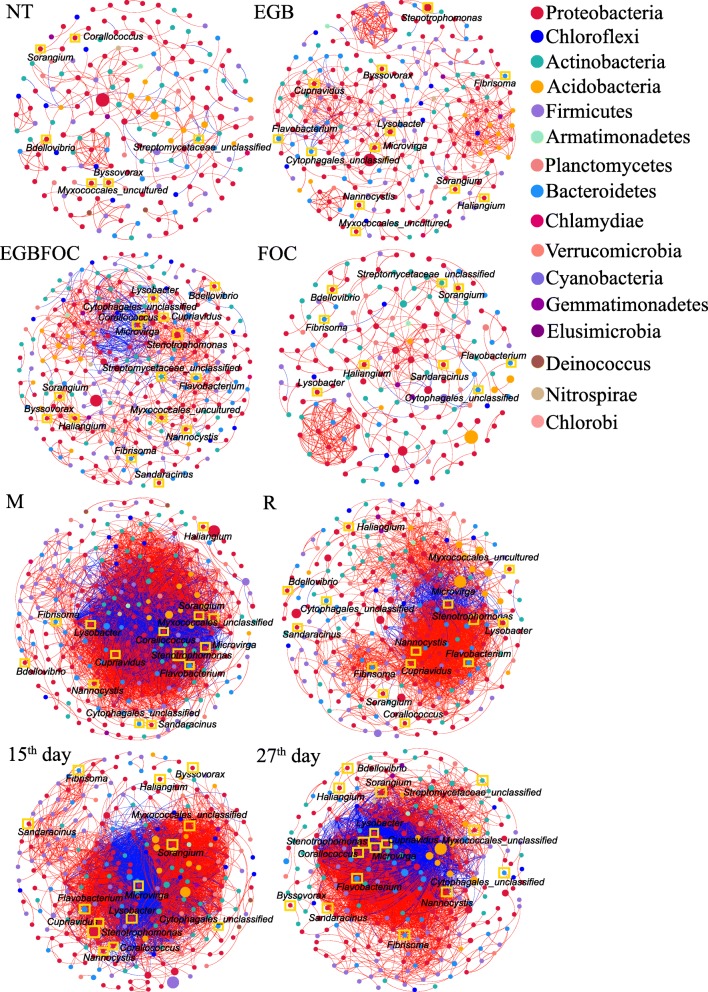
Network cooccurrence analysis (Spearman |ρ| > 0.7 and *p* < 0.001) of bacterial communities from different soil samples. Each node represents the taxonomic level of the genus (based on 16S rRNA), and the size of the node is proportional to the relative abundance of the genus in the sample. Red lines represent a positive correlation, and blue lines represent a negative correlation. Line thickness represents the degree of the correlation. Predatory bacteria are marked with yellow boxes. R, the sampling sites surrounding the roots; M, the site between the cucumber root and inoculation site; NT, no FOC or strain EGB solid culture; EGB, strain EGB solid culture only; EGBFOC, both FOC and EGB solid culture; FOC, FOC only; 15^th^ day, soils sampled on the 15^th^ day; 27^th^ day, soils sampled on the 27^th^ day

Cooccurrence network analysis was used to reveal the complexity of the connections of the microbiomes in different treatments (Spearman |ρ| > 0.7 and *p* < 0.001). Datasets were combined according to the treatments (NT, EGB, EGBFOC, and FOC), sampling sites (M, R), or sampling times (15^th^ day, 27^th^ day). The bacterial empirical cooccurrence networks differed significantly with the introduction of strains EGB and FOC into the systems as revealed by the network parameters (Table 3). The EGB treatment significantly increased the number of nodes and edges compared to the NT treatment (Fig. 6 and Table 3). FOC treatment decreased the number of nodes but increased the connectivity of the network. Notably, inoculation with both strains EGB and FOC (EGBFOC treatment) changed the node and edge numbers the most among all treatments. The number of edges increased by 3.7-fold in the EGBFOC treatment, indicating the strong interactive effects of strains EGB and FOC on the soil bacterial community. The bacterial community cooccurrence patterns of the bulk soil (M) showed similar node numbers as those of cucumber root surrounding soils (R) (Fig. 6 and Table 3) but with significantly higher edge numbers (5669 for M and 2705 for R) (Table 3). Although the number of nodes in the bacterial cooccurrence networks of the 15^th^ and 27^th^ days was not significantly different, the number of edges decreased by 7.1% from 15^th^ day to 27^th^ day (Table 3). A Venn diagram showed that a stable microbial composition occurred in all cooccurrence networks at *p* < 0.001 (Additional file 10: Fig. S7). Among all these networks, the modularities of the NT, EGB, EGBFOC, and FOC treatments were higher than 0.4 (Table 3), indicating the modular structure of the soil bacterial communities. However, the modularities of the cooccurrence networks of samples from M sites on 15^th^ day and 27^th^ day were < 0.25, and their negative edges accounted for more than 26.0% (Table 3), suggesting highly dynamic spatial and temporal community changes.

**Table 3:** Correlation and topological properties of the microbiome networks

	Bacteria								Fungi	
	NT	EGB	EGBFOC	FOC	M	R	15th day	27th day	NT + FOC	EGB + EGBFOC
Number of nodes^a^	186	252	288	174	293	279	290	283	186	158
Number of edges^b^	266	636	984	326	5669	2705	5195	4826	349	333
Positive edges^c^	238	608	804	307	3899	2390	3546	3541	289	284
Negative edges^d^	28	28	180	19	1770	315	1649	1285	60	49
Modularity^e^	0.873	0.756	0.631	0.780	0.243	0.381	0.246	0.212	0.871	0.828
Number of community^f^	39	32	29	34	19	15	21	21	29	25
Network diameter^g^	7	7	19	7	11	11	9	10	7	7
Average path length^h^	1.835	2.102	5.533	1.849	2.837	3.449	2.767	2.836	1.790	1.589
Average degree^i^	1.430	2.524	3.417	1.874	19.348	9.695	17.914	17.053	1.876	2.108
Average clustering coefficient^j^	0.175	0.208	0.186	0.218	0.288	0.254	0.292	0.264	0.270	0.286
Density^k^	0.008	0.010	0.012	0.011	0.066	0.035	0.062	0.060	0.010	0.013

Note: R, cucumber root surrounding soil samples; M, soil sampled from the site between the cucumber root and inoculation site; 15^th^ day, soils sampled on the 15^th^ day; 27^th^ day, soils sampled on the 27^th^ day; NT, no FOC or strain EGB solid culture; EGB, strain EGB solid culture only; EGBFOC, both FOC and EGB solid culture; FOC, FOC only

^a^Microbial taxa (at the genus level) with at least one significant (*p* < 0.001) and strong (*r* > 0.7 or ≤ 0.7) correlation. R language and corr.test() were used for correlation analysis

^b^Number of connections obtained by R language analysis (R 2017, ^4^version 3.5.3)

^c^Positive correlation (> 0.7 with *p* < 0.01) between two microbial taxa

^d^Negative correlation (≥ 0.7 with *p* < 0.01) between two microbial taxa

^e^Structure with high-density connections between nodes (inferred by Gephi)

^f^A community is defined as a group of nodes that are densely connected internally (Gephi)

^g^The longest distance between nodes in the network, measured in number of edges (Gephi)

^h^Average network distance between all pair of nodes or the average length of all edges in the network (Gephi)

^i^The average number of connections of every node in the network (Gephi)

^j^The average clustering coefficient is defined as the mean value of individual coefficients (Gephi)

^k^The density used to measures how close the network is to complete. A complete graph has all possible edges and density equal to 1 (Gephi)

Compared with the control, agglomeration of the cooccurrence networks was found after application of the strain EGB solid culture and FOC. Maximum connectivity was observed in the EGBFOC treatment with an edge degree of 34 for the keystone taxon *Acetobacteraceae*_unclassified and an average degree of 3.417, compared to 9 and 1.430 for the NT treatment, respectively (Additional file 11: Table S4 and Table 3). Agglomeration of cooccurrence networks was observed in all communities considering the treatment and spatiotemporal distribution, whereby 2 clusters connected by large numbers of negative edges were observed (Fig. 6). Seventeen potential predatory bacteria [32, 33] were found in the cooccurrence networks, eight of which were myxobacteria (listed in Additional file 12: Table S5), accounting for 3.8%, 4.8%, 5.9%, and 5.2% of the total nodes in the NT, EGB, EGBFOC, and FOC treatments, respectively. Predatory bacteria showed intensive connections with other node taxa in the networks (Additional file 12: Table S5 and Additional file 13: Table S6). The relative abundances of *Corallococcus*, *Nannocystis*, *Flavobacterium*, *Stenotrophomonas*, *Cupriavidus*, *Lysobacter*, and *Microvirga* increased significantly following the EGB solid culture treatments (EGB and EGBFOC) compared to the treatments without EGB solid culture (NT and FOC) (*p* < 0.05, Additional file 14: Table S7). Several predatory bacteria appeared as hubs or indicators of the networks (Additional file 11: Table S4 and Additional file 12: Table S5), underscoring their important roles in shaping the soil bacterial communities. Although the properties of fungal networks were not significantly changed after strain EGB solid culture application, the number of nodes in the fungal network was decreased significantly (Additional file 15: Fig. S8 and Table 3).

## Discussion

Myxobacteria are promising potential biocontrol agents as revealed in greenhouse experiments, providing effective control of damping-off disease of tree seedlings in sterile peat or soil [34, 35], cucumber Fusarium wilt [13], and anthracnose in hot peppers [36, 37]. However, field trials have not been conducted to evaluate the myxobacterial biocontrol of soil-borne fungal pathogens of vegetable crops. Strain EGB solid culture effectively decreased the wilt disease incidence in cucumber field trials. qPCR analysis indicated that strain EGB was well adapted to the soil environment. The strain EGB quantity in cucumber root surrounding soil remained at a level of 10^5^–10^6^ copies g^–1^ soil at the end of the experiments, which is a critical level for BCAs to protect plants [38]. The induction of *Corallococcus* by FOC was observed, indicating the interaction between myxobacteria and FOC. However, its quantity was 1.1–1.6 orders of magnitude lower than those in strain EGB treatments.

Effective colonization and formation of biofilm-like structures on plant roots are crucial for the biocontrol of plant diseases by plant growth-promoting rhizobacteria (PGPR) [38–40]. The enhanced biofilm formation in *Pseudomonas fluorescens* CHA0 significantly enhanced the colonization of carrot roots and led to more stable interactions between mixed inocula and the rhizosphere [41]. Strain EGB colonized the root elongation zone and displayed visible biofilm-like structures in hydroponic experiments. Myxobacteria secrete abundant extracellular matrices, which can facilitate biofilm formation and root colonization [20]. The ability of myxobacteria to feed on other soil microbes leads to the propagation of these bacteria and reduction in the quantity of their prey. However, traditional BCAs have to compete for resources with the native microbial community to survive in the soil, which reduces their adaptive abilities.

Movement of bacteria in soil strongly affects their colonization efficiency, microbial fitness, and bacteria-mediated biocontrol abilities [42, 43]. Active movements can be driven by flagella in the form of chemotaxis or swarming. Intact chemotaxis and swarming motility machineries were found to be important for PGPR to reach and colonize plant roots [44, 45]. Hypermotility mutations improved the colonization of alfalfa root surfaces by *P. fluorescens* F113 and improved its biocontrol activity [46]. Gao et al. further showed that swarming played an important role in root colonization by *B. subtilis* [45]. However, movement driven by flagella was limited in wet soil [47]. Vigorous movement of strain EGB towards cucumber roots was revealed by spatiotemporal high-throughput sequencing. A movement of 8 cm from the inoculation site to the roots was observed for strain EGB. Although myxobacteria lack flagella and are unable to swim in liquid environments, these organisms glide on solid surfaces in coordinated cell groups or as isolated individuals. This gliding motility driven by a focal adhesion complex allows myxobacterial cells to glide over relatively dry and hard soil matrices [48].

We speculate that root exudates of cucumbers attracted the movement of strain EGB in the soil. Many studies have shown that chemotaxis towards root exudates is the first step of bacterial colonization of roots [49]. Organic acids, amino acids, sugars, quaternary ammonium salts, and secondary metabolites secreted by plants are all potential chemoattractants for beneficial bacteria [49–52]. Our results showed that strain EGB could also be attracted by root exudates. Detailed analysis indicated that several compounds in the root exudates induced chemotactic movement of strain EGB. Maltose and maltitol were the most efficient chemoattractants, attracting strain EGB at low concentrations (10 μM). Maltose has been determined to be an important compound in the root exudates of cucumbers [49, 53] and other plants [54]. Although chemotaxis of myxobacteria towards small soluble chemicals is doubtful [55, 56], chemotaxis driven by pili-based single-cell “twitching motility” has been shown to be important in the biofilm development of *Pseudomonas aeruginosa* [57]. This research identified the chemotaxis of myxobacteria towards a sugar from root exudates, which has not been reported before and may play important roles in the colonization of cucumber roots by strain EGB. The role of maltose in the myxobacteria-root interaction deserves further investigation in future studies.

The rhizosphere microbiome is important for plant growth, nutrition, and health. Disease-suppressive soils offer the best examples for the protection of crop plants against infection by soil-borne pathogens [15]. The development of suppressive soils can be induced by long-term monoculture or organic amendments [8]. Unique bacterial communities have been detected in suppressive soils [12, 58]. Detailed analysis showed that specific bacterial strains played roles in the suppression of soil-borne diseases [59, 60]. *Pseudomonas* and *Streptomyces* isolated from suppressive soils were shown to produce antibiotics that inhibit Rhizoctonia damping-off in potato and Fusarium wilt in strawberry [59, 60]. However, many microbial species isolated from suppressive soils failed to establish or survive in soil or on plant roots because of competition with the indigenous soil microbiome. Reintroduction of antagonistic strains modified the plant microbiota slightly or transiently and has met with limited success in large-scale agricultural applications [15, 61]. Bacterivorous protists have been shown to be a central hub in the soil microbiome [62] and contributed to the rarity of certain bacterial taxa in the field [63].

As a predatory bacterium, strain EGB is able to prey on a variety of bacteria. Therefore, we deduced that strain EGB may affect the soil microbiota. Community structure analysis showed that strain EGB dynamically changed the soil bacterial diversity. Cooccurrence network analysis indicated that strain EGB drove the alteration of microbial communities. A highly connected root surrounding microbiome was found to decrease pathogen invasion success [64–66]. Significant positive correlations between potential plant-beneficial bacteria, such as *Bacillus*, *Pseudomonas*, *Azotobacter*, and *Lysobacter*, and predatory bacteria were observed (Additional file 13: Table S6). The positive correlation between *Corallococcus* and other genera of potentially beneficial bacteria might contribute to the protection against fungal infection, but need further verification (Additional file 11: Table S4).

The interaction networks of fungal communities after EGB solid culture application became more fragile (Additional file 15: Fig. S8 and Table 3). Although it remains unclear whether the reduction of soil fungal diversity is an indicator of the suppressive process, many studies have suggested that the fungal diversity and the complexity of the fungal community structure are negatively correlated with disease suppression [64, 67–69].

The various modes of action of BCAs have been studied in detail. However, successful biocontrol depends not only on plant-microbe interactions but also on the ecological fitness of the BCAs [3]. Cell densities of artificially introduced PGPR always declined to a low level within a few weeks, while a crucial colonization level must be reached to implement biocontrol function [38]. However, at low-cell densities, it is difficult for BCAs to accumulate enough antibiotics to act against pathogens, to occupy ecological niches, or to compete for nutrition with soil-borne pathogens. In addition to the direct impact of BCA strains on phytopathogens, the effects of BCA-driven modification of the microbial community on pathogen development are now being considered [61]. We believe that biocontrol by predation offers a great opportunity for the management of soil-borne plant pathogens. Direct predation of phytopathogens and competing soil bacteria is also desirable for the adaptability of BCAs in the soil environment [70]. Myxobacteria are slow-growing bacteria with the ability to produce heat-tolerant spores, and slow-growing, heat-tolerant bacterial families are proposed to have potential roles in plant protection [71]. Notwithstanding, the nontargeted effect of strain EGB on soil microbiota should be given more attention in future research due to its broad prey spectrum.

## Conclusions

The importance of the rhizosphere microbiome in plant resistance to soil-borne pathogens has been widely recognized, while predatory microbes have received less attention for the biocontrol of phytopathogens. The predatory myxobacterium, *Corallococcus* sp. strain EGB, showed a strong biocontrol effect against Fusarium wilt disease in field trials. Our results indicate that strain EGB modified the soil microbial community structure and reduced the quantities of FOC in the soil by predation. Considering the predatory habits of strain EGB and its well adaption to the soil environment, strain EGB has great potential to be commercially developed as a novel biocontrol agent for Fusarium wilt.

## Materials and methods

### Microbial strains and growth conditions

*Fusarium oxysporum* f. sp. *cucumerinum* (FOC) (ACCC 30220), *Corallococcus* sp. strain EGB (CCTCC M2012528), and *Escherichia coli* DH10B (ThermoFisher Scientific, USA) were maintained in the laboratory at – 80 °C with 15.0% glycerol (Sigma-Aldrich, Japan) as a cryoprotectant. To prepare conidia, FOC was cultured on potato dextrose agar (PDA) plates [72] for 3 days at 28 °C, after which 5-mm-diameter mycelial plugs were cut from the periphery of the colonies and used to inoculate 250-ml flasks containing 100 ml of PDB medium [72]. The flasks were incubated at 28 °C for 5 days. A conidial suspension was obtained by filtering the liquid culture through three layers of sterile gauze to remove the mycelia. The conidia were examined using phase-contrast microscopy to ensure a spore purity of > 95.0%, and the concentration was determined using a hemocytometer (XB-K-25, Qiujing, China). To prepare inocula for solid-state fermentation, strain EGB was incubated on VY/4 agar medium [25] for 3 days and then transferred into test tubes (φ20 mm × 150 mm) containing 4 ml of LBS medium (5 g of yeast extract, 1 g of tryptone, 7 g of soluble starch, and 1 L of water). After 24 h of incubation, strain EGB was transferred into a 250-ml flask containing 100-ml of LBS medium and incubated for 24 h.

In the root colonization experiment, strain EGB was routinely grown in CTT medium [73] at 30 °C for 12 h and then centrifuged and resuspended in TPM buffer [74] to an OD_600_ of 1.0. *E. coli* DH10B was grown in LB broth for 12 h on a shaker, then centrifuged at 10 000×*g* for 10 min and resuspended in TPM buffer to an OD_600_ of 1.0. Strains EGB and DH10B were cultivated at 30 °C and 37 °C, respectively. All liquid cultivations were performed in a rotary shaker (IS-RDS4, CRYSTAL, USA) at 180 rpm.

### Predation experiments

Strain EGB and its bacterial prey (Additional file 16: Table S8) were cultured in LBS and LB medium to an OD_600_ of 1.0. Then, the bacterial cells were collected by centrifugation at 10 000×*g* for 3 min and resuspended in sterile H_2_O to 10^9^ CFU (colony forming units) ml^−1^. An aliquot of 200 μl of bacterial prey cell suspension was dropped onto a TPM plate, and 3 μl of strain EGB was dropped into the center of the prey cells and cultivated for 48–60 h. The heat-inactivated strain EGB was used as a control. The number of surviving prey cells was counted by serial dilution on LB agar plates, on which strain EGB did not grow. All bacteria were cultured at 30 °C.

### Preparation of strain EGB solid culture

The matrix used for solid-state fermentation of EGB was composed of rice straw and rabbit dung, both of which were ground and mixed at a ratio of 1:2. The final substrate contained 1.7% nitrogen (N), 0.7% P_2_O_5_, and 0.6% K_2_O. Rice straw was obtained from the countryside of Xinyang (China), and rabbit dung was purchased from Haoyun Ltd. (China). The solid-state fermentation was performed in trays. The water content of the matrix was 55.0–60.0%. The solid matrix was sterilized for 30 min at 121 °C for solid-state fermentation. Strain EGB was sprayed onto the sterile matrix at a rate of 100 ml per kilogram using the inoculum as described above, mixed thoroughly, and incubated at 30 °C for 7 days. The cell density of strain EGB in the solid culture was determined by qPCR. The primers for the detection of strain EGB were EGBF (5′-TCAT CATCGGCACTGTCATC-3′) and EGBR (5′-GGATGGTGCGGTTGAGGAGC-3′). The solid culture contained approximately 1 × 10^9^ CFU of strain EGB g^−1^ dry matrix.

### Soil

The soil used for the greenhouse experiments was collected from a field in Xiamafang (118° 50′ E, 32° 2′ N), Nanjing, China. The collection field had never been planted with cucumber. The soil properties were as follows: pH 6.63, organic matter 39.2 g kg^−1^, available N 21.01 mg kg^−1^, available P 42.7 mg kg^−1^, available K 194 mg kg^−1^, total N 1.90 g kg^−1^, total P 0.767 g kg^−1^, and total K 14.1 g kg^−1^. The soil was homogenized by passage through a 6-mm sieve to remove roots, stones, and other residues before it was used in the pot experiments.

### Greenhouse experiment

Cucumber (*Cucumis sativus* L.) seeds of the “Jinchun 4” cultivar were purchased from the Jiangsu Academy of Agricultural Sciences (Nanjing, China). The cucumber seeds were pretreated and germinated under suitable conditions as previously reported [9]. The pot experiments were divided into four groups as follows: NT, no treatment (no FOC pathogen or strain EGB solid culture); EGB, strain EGB solid culture treatment; EGBFOC, strain EGB solid culture and FOC pathogen treatment; and FOC, FOC pathogen treatment only. There were 7 pots (φ22 cm × 15 cm) in each treatment and 3 cucumber plants in each pot. Each plant was marked, and one cucumber was selected from each pot to form a repeat. Thus, 21 cucumbers were divided into three repeats, and every repeat contained 7 cucumbers. Each pot contained 3 kg of soil, and seedlings with three true leaves were transplanted. FOC was inoculated at a concentration of 10^5^ conidia g^-1^ soil using the conidia described above. Strain EGB solid culture was applied at 15 g pot^-1^ in the center of the pot. Pots were labeled and randomly placed in the greenhouse to minimize the influence of light and temperature differences. The experiment was conducted from May 29 to June 25 of 2015 with temperatures of 30–35 °C and relative humidity ranging from 65.0 to 80.0%. The disease symptoms of the cucumbers were observed and recorded every 2 weeks. Cucumber symptoms were divided into 4 levels as follows [75]: 0, the cucumber plant was healthy; 1, < 25.0% of leaves were wilted; 2, 25.0 to 50.0% of leaves were wilted; 3, 50.0 to 75.0% of leaves were wilted; and 4, > 75.0% of leaves were wilted. The disease index for every treatment was calculated according to the following formula: disease index = [∑(rating × number of plants rated)/(total number of plants × highest rating)] × 100. Statistical analysis of the disease incidence and the populations of strains FOC and EGB in the soil surrounding the roots were conducted at the end of the experiment. Soil samples were collected every 2 weeks, and the soil sampling method is described below.

### Biocontrol of cucumber Fusarium wilt in field trials

The field trials were conducted in Changshu (120° 98′ E, 31° 66′ N), Jiangsu Province of China, where cucumbers had been continuously monocropped for more than 3 years. In 2015, the biocontrol experiment was divided into three groups as follows: control without any treatment, treatment with strain EGB liquid culture, and treatment with EGB solid culture. A total of 200 cucumber plants in each treatment were randomly divided into four blocks. The trial was carried out 2 weeks after seedlings were transplanted into the field. The liquid-fermented culture of strain EGB was administered to the roots at 25 ml per plant, and the EGB solid culture was placed in a hole in the soil near the cucumber roots at 10 g per plant. Trials in 2016 were set up similar to those in 2015, except that sterilized EGB solid culture was used instead of strain EGB liquid culture. The disease incidence was evaluated and statistically analyzed for the greenhouse experiment after 1 month.

### Analysis of root colonization by *Corallococcus* sp. strain EGB

The colonization of cucumber roots by strain EGB was performed in a modified hydroponic system [29] with *E. coli* DH10B as a control [31], which were prepared as described above. Cucumber seeds of “Jingchun 4” were surface-sterilized with 2% NaClO solution for 15 min, then washed with sterile distilled water, and planted into aseptic tissue culture bottles containing vermiculite. The cucumber seeds were germinated and grown in the growth chamber at 23 °C with a 16-h light regimen for 4 days. Then, the seedlings were aseptically transplanted into 50 ml flasks containing 35 ml of sterile 1/4 sucrose-free Murashige Skoog medium [76], and incubated in a climate-controlled incubator (GXZ-500B, Ningbo Southeast Instrument CO., LTD, China) at 30 °C with the same photoperiod until the plants have grown four true leaves. The hydroponic system was checked for their sterility by taking a 100-μL aliquot and spreading it on solid LB plates, and the contaminated plants were discarded. Finally, axenically prepared cucumber seedlings were gently washed with sterile distilled water and placed into a 250-ml flask containing 100 ml of a suspension of either strain EGB or *E. coli* DH10B. The flasks were incubated in a climate-controlled incubator with the same conditions for 2 days. After incubation, the cucumber roots were gently washed with sterile distilled water, cut into 0.5–1 cm pieces, and fixed in 2.5% glutaraldehyde solution for 2 h. The root pieces were washed thrice for 10 min in 25-mM sodium phosphate buffer, then they were serially dehydrated in 50%, 70%, 80%, and 90% acetone solutions for 15 min, respectively, and finally in 100% acetone for 90 min, during this process, the acetone was changed every 30 min. Cucumber root pieces were transferred into *tert*-butyl alcohol to displace acetone thrice for 30 min each time, and then freeze-dried in a Heto Power Dry LL3000 freeze dryer (Thermo Scientific, Waltham, MA, USA). These samples were then coated with gold (~ 10 nm) using an ion-sputter coater (E-1010, Hitachi, Japan). Finally, the root samples were observed by scanning electron microscopy (SU8010 SEM, Hitachi, Japan). Each treatment was repeated three times.

### Assessment of chemotaxis towards cucumber root exudates

The root exudates were collected and processed as reported in a previous study [40]. After collection, the root exudates were filtered through a 0.22-μm membrane (Millipore, Merck KGaA, Germany), freeze-dried, and then stored at – 80 °C for the chemotaxis assay.

A modified method based on the Petri-plate assay [55] was used for the chemotaxis assay. Semisolid 1/10 CTT medium (0.1% casitone, 10 mM Tris-HCl [pH 7.6], 8 mM MgSO_4_, 1 mM KH_2_PO_4_, and 0.5% agar) was poured into 6 cm sterile plastic plates, and 3 μl of strain EGB suspension prepared as above was spotted onto each plate 2–4 mm away from the root exudates. Then, the plates were incubated in the dark at 30 °C for 24 h, and the swarming of strain EGB was observed and recorded with a stereoscopic microscope (Nikon SMZ-10) every 6 h. The chemotactic responses of strain EGB towards 13 substances detected in cucumber root exudates [29, 30] were further analyzed. The substances were as follows: butanedioic acid, citric acid, fumaric acid, malic acid, maltitol, sorbitol, arabitol, tryptophan, tyrosine, alanine, aspartic acid, valine, maltose, and glucose.

### Soil sampling and DNA extraction

To investigate the movement of strain EGB and its effects on the soil microbial community, two sampling sites were set; the M site, which was located in the middle of the cucumber root and the EGB solid culture inoculation site (I), and the soil surrounding the cucumber roots (R) (Fig. 2a). On the 15^th^ and 27^th^ days, for each treatment, 9 samples of R or M sites were randomly selected from 3 replicates, and the 3 soils from the same replicate were mixed as one composite sample. Aliquots comprising 5 g soils were collected at every point at a depth of 5–6 cm. Cucumber roots, stones, and visible residues were removed by passing the samples through a 2-mm sieve. Finally, the fresh soil samples were stored at – 80 °C before DNA extraction and microbial community analysis.

Total DNA was extracted from 0.5-g soil samples using the FastDNA® SPIN Kit for soil (MP Biomedicals, Santa Ana, CA) and purified using the DNA Clean-Up^TM^ kit (MO BioLabs, Solana Beach, CA, USA). The prepared DNA was used as a template in the following PCR amplification.

### Real-time qPCR

The relative quantities of FOC and strain EGB in the soil surrounding the roots of the cucumbers were determined by real-time qPCR amplification, which was performed with an Applied Biosystems 7500 Real-Time PCR system (Applied Biosystems, USA) using a Premix Ex Taq™ kit (Takara, Dalian, China). The primers FocF3 (5′-AAAC GAGCCCGCTATTTGAG-3′) and FocR7 (5′-TATTTCCTCCACATTGCCATG-3′) were used for the detection of FOC [9], and the primers EGBF (5′-TCATCATCGGCACTGTCATC-3′) and EGBR (5′-GGATGGTGCGGTTGAGGAGC-3′), targeting a single copy TonB-dependent receptor gene, which were designed by comparative analysis of the strain EGB genome and that of other myxobacteria, were used for the detection of strain EGB. Real-time PCR amplification reactions were performed in 20 μl volumes containing 20 ng of template DNA, 0.4 μl of each primer (10 μM), 10 μl of Premix Ex Taq™ (2×), 0.4 μl of ROX Reference Dye II (×50), and 6.8 μl of double-distilled water. The PCR comprised 30 s at 98 °C, followed by 40 cycles of 5 s at 95 °C and 34 s at 60 °C. Standard curves were generated as described previously [9]. Agarose gel electrophoresis and melting curve analysis were used to indicate the specificity of the amplification products.

### Sequencing using MiSeq PE300

The V4–V5 hypervariable regions of the bacterial 16S rRNA gene were amplified using the primers 515F (5′-CCTACGGGAGGCAGCAG-3′) and 907R (5′-TTA CCGCGGCTGCTGGC-3′). The primers ITS1F (5′-GGTTTCTGTAGGTGAACC TGC-3′) and ITS2R (5′-CTCGGACGAGGATCCTCGCC-3′) were used to amplify the ITS1-ITS2 hypervariable region of fungi. All PCRs were carried out as previously reported [77]. The PCR products were purified using a PCR Clean-Up^TM^ kit (MO BioLabs, Solana Beach, CA, USA) and sent to the Majorbio Company (Shanghai, China) for sequencing.

After quality assessment, the amplicons were sequenced by an Illumina MiSeq PE300. After sequencing, the raw sequences were processed using Trimmomatic [78], and the final sequences were assembled using paired-end reads after overlapping using Flash molecular editor [79]. Then, the assembled reads were assigned to operational taxonomic units (OTUs) using the Usearch software platform (version 7.1 http://qiime.org/) with 97 % cut-off [80]. The Uchime software (version 4.2.40) was used to remove the chimeric OTUs [81]. Finally, the bacterial representative sequences and the fungal OTUs were matched against the RDP [82] and UNITE databases [83], respectively. The relative abundance of *Corallococcus* was calculated as the ratio of *Corallococcus* OTU to the total OTUs in a sample.

Because the data did not conform to the assumptions of the general linear model, the correlation between two genera in cooccurrence analyses of different groups was calculated using the Spearman package of R (Version 3.5.3), and network visualization was carried out by Gehpi (Spearman |ρ| > 0.7 and *p* < 0.001) following the previously described method [64]. The degree of each genus was calculated using Gehpi, and keystones and hubs were defined as the top 1% and 2% of degrees in every group, respectively. Indicators (at the genus level) of different groups were determined using the indicspecies package of R (Version 3.5.3) at different levels (stat > 0.90, *p* < 0.01 in NT, EGB, EGBFOC, and FOC groups; stat > 0.95, *p* < 0.01 in M, R, day 15, and day 27 groups).

### Statistical analyses

One-way analysis of variance (ANOVA) was performed to evaluate the differences between the four treatments in the pot experiment. Based on the high-throughput sequencing results, the Mothur program (version v.1.30.1) [77] was used to draw the rarefaction curve and Shannon index curve, as well as to determine the richness estimators and diversity indices. PCA was conducted using the rda () function in the vegan package of R (Version 3.5.3, vegan package). R (Version 3.5.3, Venn Diagram package) was employed to generate the Venn diagram. Duncan’s multiple-range test was performed if significant differences (*p* < 0.05) were indicated for the results by one-way ANOVA. All statistical analyses were performed in SPSS 13.0 (IBM Corp., USA).

## Supplementary information

**Additional file 1: Table S1.** Detection of the number of myxospores in strain EGB solid culture at different storage times.

**Additional file 2: Figure S1.** Utilization of maltose by strain EGB.

**Additional file 3: Figure S2.** Predation effect of *Corallococcus* sp. EGB on bacteria. Strain EGB and other bacteria were cultured in LBS and LB medium, respectively, until reaching an OD_600_ of 1.0. Then, the EGB and other bacteria were collected by centrifugation at 10 000 × g for 3 min and resuspended in sterile dH_2_O to 10^9^ CFU/ml. An aliquot comprising 200 μl of each bacterial suspension was dropped onto TPM plates, and 3 μl of the strain EGB suspension was added to the center of the prey colonies. An activated EGB suspension was used as a control. The number of survivors in every colony was assessed by gradient dilution and plate counting after 36 hours of culture on the plate at 30°C.

**Additional file 4: Table S2**. Sample list and sequencing information of the 16S_V4–V5_ gene libraries.

**Additional file 5: Figure S3.** Rarefaction curves of bacteria depicting the effect on the number of OTUs identified at 97% similarity. A. The soil samples were collected on the 15^th^ day. B. The soil samples were collected on the 27^th^ day. R, the sampling sites surrounding the roots; M, intermediate site between the cucumber root and inoculation site; NT, no FOC or strain EGB solid culture; EGB, strain EGB solid culture only; EGBFOC, both FOC and EGB solid culture; FOC, FOC only.

**Additional file 6: Figure S4.** Comparison of bacterial diversity indices between different treatments. A, B, C, D, and E represent the changes in different diversity indices based on the 16S_v4-v5_ gene among different treatments. R, the sampling sites surrounding the roots; M, intermediate site between the cucumber root and the inoculation site; 15, soils sampled on the 15^th^ day; 27, soils sampled on the 27^th^ day; NT, no FOC or strain EGB solid culture; EGB, strain EGB solid culture only; EGBFOC, both FOC and EGB solid culture; FOC, FOC only. Bars indicate the standard deviations of the averages from three replicates. Columns with different letters are significantly different at *p* ≤ 0.05 according to Duncan’s test.

**Additional file 7: Table S3.** Sample list and sequencing information of the 18S_ITS1-ITS2_ gene libraries.

**Additional file 8: Figure S5.** Rarefaction curves for the number of fungal OTUs with different treatments at 97% similarity. R, the sampling sites surrounding the roots; 27, soils sampled on the 27^th^ day; NT, no FOC or strain EGB solid culture; EGB, strain EGB solid culture only; EGBFOC, both FOC and EGB solid culture; FOC, FOC only.

**Additional file 9: Figure S6.** Comparison of fungal diversity indices between different treatments. R, the sampling sites surrounding the roots; 27, soils sampled on the 27^th^ day; NT, no FOC or strain EGB solid culture; EGB, strain EGB solid culture only; EGBFOC, both FOC and EGB solid culture; FOC, FOC only. Bars indicate the standard deviations of the averages from three replicates. Columns with different letters are significantly different at *p* ≤ 0.05 according to Duncan’s test.

**Additional file 10: Figure S7.** The common and exclusive bacterial genera in the cooccurrence network (at *p* < 0.001) of different treatments (A), sampling sites (B) and sampling times (C) are shown by Venn diagrams. NT, no FOC or strain EGB solid culture; EGB, strain EGB solid culture only; EGBFOC, both FOC and EGB solid culture; FOC, FOC only; R, the sampling sites surrounding the roots; M, intermediate site between the cucumber root and the inoculation site; day 15, soils sampled on the 15^th^ day; day 27, soils sampled on the 27^th^ day.

**Additional file 11: Table S4.** Analysis of the correlation between *Corallococcus* and keystones (Spearman |ρ| > 0.7, *p* < 0.001, top 1% degree in cooccurrence network), hubs (Spearman |ρ| > 0.7, *p* < 0.001, top 2% degree in cooccurrence network) and indicators. Note: NT, no FOC or strain EGB solid culture; EGB, strain EGB solid culture only; EGBFOC, both FOC and EGB solid culture; FOC, FOC only; R, the sampling sites surrounding the roots; M, intermediate site between the cucumber root and the inoculation site; day 15, soils sampled on the 15^th^ day; day 27, soils sampled on the 27^th^ day.

**Additional file 12: Table S5.** Abundance and degree analysis of predatory bacteria in the cooccurrence network (Spearman |ρ| > 0.7, *p* < 0.001). Note: At the Spearman |ρ| > 0.7 and *p* < 0.001 level, predatory bacteria that do not appear in the cooccurrence network are shown in blue text. NT, no FOC or strain EGB solid culture; EGB, strain EGB solid culture only; EGBFOC, both FOC and EGB solid culture; FOC, FOC only; R, the sampling sites surrounding the roots; M, intermediate site between the cucumber root and the inoculation site; day 15, soils sampled on the 15^th^ day; day 27, soils sampled on the 27^th^ day.

**Additional file 13: Table S6.** Analysis of correlation between predatory bacteria and other microorganisms (Spearman |ρ| > 0.7, *p* < 0.001). Note: At the Spearman |ρ| > 0.7 and *p* < 0.001 level, predatory bacteria that do not appear in the cooccurrence network are shown in blue text. NT, no FOC or strain EGB solid culture; EGB, strain EGB solid culture only; EGBFOC, both FOC and EGB solid culture; FOC, FOC only; R, the sampling sites surrounding the roots; M, intermediate site between the cucumber root and the inoculation site; day 15, soils sampled on the 15^th^ day; day 27, soils sampled on the 27^th^ day.

**Additional file 14: Table S7.** Statistical analysis of the relative abundance of predatory bacteria in different treatment groups. Note: NT, no FOC or strain EGB solid culture, EGB, strain EGB solid culture only; EGBFOC, both FOC and EGB solid culture; FOC, FOC only. Data were expressed as the means ± standard deviations in each treatment. In the same row, values followed by the same letters are not significantly different at *p* ≤0.05 according to Duncan’s test.

**Additional file 15: Figure S8.** Network cooccurrence analysis of fungal communities in soil samples with different treatments. To elucidate the effect of strain EGB solid culture on the changes in the soil fungal community, the NT treatment and FOC treatment were classified into group (A) and group (B) comprising the EGB treatment and the EGBFOC treatment. R language (R 2017, 4 version 3.3.3) was run using the R-studio environment, and corr.test() was used for correlation analysis. A connection represents correlations with magnitude>0.7 (positive correlation-red edges) or <− 0.7 (negative correlation-blue edges) that are statistically significant (*p* < 0.01). Each node represents the taxonomic level of the genus (based on ITS1-ITS2 rRNA), and the size of a node is proportional to the relative abundance of the genus in the sample. Red lines represent a positive correlation, and blue lines represent a negative correlation. Line thickness represents the degree of correlation.

**Additional file 16: Table S8.** Bacterial prey species used in the predation experiments. Note: Strains were maintained in our laboratory at -80°C with 15% glycerol (Sigma, Japan) as a cryoprotectant.

**Additional file 17: Table S9.** Corresponding sample names in the manuscript and in NCBI depository.

**Additional file 18:** Image of Figure 6 with information of other microorganisms.

## Data Availability

The datasets generated and analyzed during the current study are available in the NCBI Sequence Read Archive (SRA) repository under the BioProject with accession codes PRJNA414260 (bacterial data) (https://www.ncbi.nlm.nih.gov/bioproject/?term=PRJNA414260) and PRJNA414050 (fungal data) (https://www.ncbi.nlm.nih.gov/bioproject/?term=PRJNA414050). To facilitate a better understanding of the experimental design and to annotate the legends more clearly, we replaced the original sample names with the new ones in the manuscript (Additional file 17: Table S9).

## Abbreviations

FOC: *Fusarium oxysporum* f. sp. *cucumerinum*
BCAs: Biological control agents
PCA: Principal component analysis
SEM: Scanning electron microscopy
PGPR: Plant growth-promoting rhizobacteria
