# Supplementary material for: A predatory myxobacterium controls cucumber Fusarium wilt by regulating the soil microbial community
Source: Microbiome. 2020 Apr 6;8:49. doi: 10.1186/s40168-020-00824-x (PMC7137222; doi:10.1186/s40168-020-00824-x)
Supplement: Supplementary file 2 — Additional file 1: Table S1. Detection of the number of myxospores in strain EGB solid culture at different storage times. [file 40168_2020_824_MOESM1_ESM.docx]

**Table S1** Detection of the number of myxospores in strain EGB solid culture at different storage times.

| Time (d) | 0 | 30 | 60 | 90 | 150 | 200 | 280 | 360 | 420 |
| --- | --- | --- | --- | --- | --- | --- | --- | --- | --- |
| Number of myxospores (Log_10_ CFU/g) | 7.92±0.04^a^ | 7.37±0.15^b^ | 7.36±0.29^b^ | 7.47±0.06^b^ | 8.01±0.18^a^ | 7.21±0.37^bc^ | 7.48±0.04^b^ | 6.93±0.24^c^ | 7.12±0.15^bc^ |

Note: 0.4 g strain EGB solid culture was taken out from the storage bag and followed by ultrasonic disruption and heat treatment as described previously [[1](#_ENREF_1)]. The number of the myxospores was measured on VY/4 medium [[2](#_ENREF_2)] after gradient dilution. The data represent the means and standard deviations (mean ± SD) from three replications. Values designated with the same letters were not significantly different (p ≤ 0.05) according to Duncan’s test.

1. Gronewold TM, Kaiser D. The act operon controls the level and time of C‐signal production for Myxococcus xanthus development. Mol Microbiol. 2001;40(3):744-56.

2. Li Z, Ye X, Liu M, Xia C, Zhang L, Luo X, et al. A novel outer membrane β-1, 6-glucanase is deployed in the predation of fungi by myxobacteria. The ISME J. 2019.
