# Supplementary material for: A predatory myxobacterium controls cucumber Fusarium wilt by regulating the soil microbial community
Source: Microbiome. 2020 Apr 6;8:49. doi: 10.1186/s40168-020-00824-x (PMC7137222; doi:10.1186/s40168-020-00824-x)
Supplement: Supplementary file 3 — Additional file 2: Figure S1. Utilization of maltose by strain EGB. [file 40168_2020_824_MOESM2_ESM.docx]

**Figure. S1** Utilization of maltose by strain EGB. Strain EGB was inoculated into 250 ml flask containing 100 ml liquid medium (0.3% maltose, 0.3% (NH4)_2_SO_4_, 10 mM Tris-HCl [pH 7.6], 8 mM MgSO4, 1 mM KH2PO4) and incubated at 30°C for 48 hours. The total sugar and reducing sugar in the liquid culture were determined every 12 h by the anthrone method [[1](#_ENREF_1)] and the dinitrosalicylic (DNS) acid method [[2](#_ENREF_2)], respectively.

1. Fales FW. The assimilation and degradation of carbohydrates by yeast cells. J Biol Chem. 1951;193(1):113-24.

2. Miller GL. Use of Dinitrosalicylic Acid Reagent for Determination of Reducing Sugar. Anal Biochem. 1959;31(3):426-8.
