# Supplementary figures and images for: A predatory myxobacterium controls cucumber Fusarium wilt by regulating the soil microbial community
Source: Microbiome. 2020 Apr 6;8:49. doi: 10.1186/s40168-020-00824-x (PMC7137222; doi:10.1186/s40168-020-00824-x)

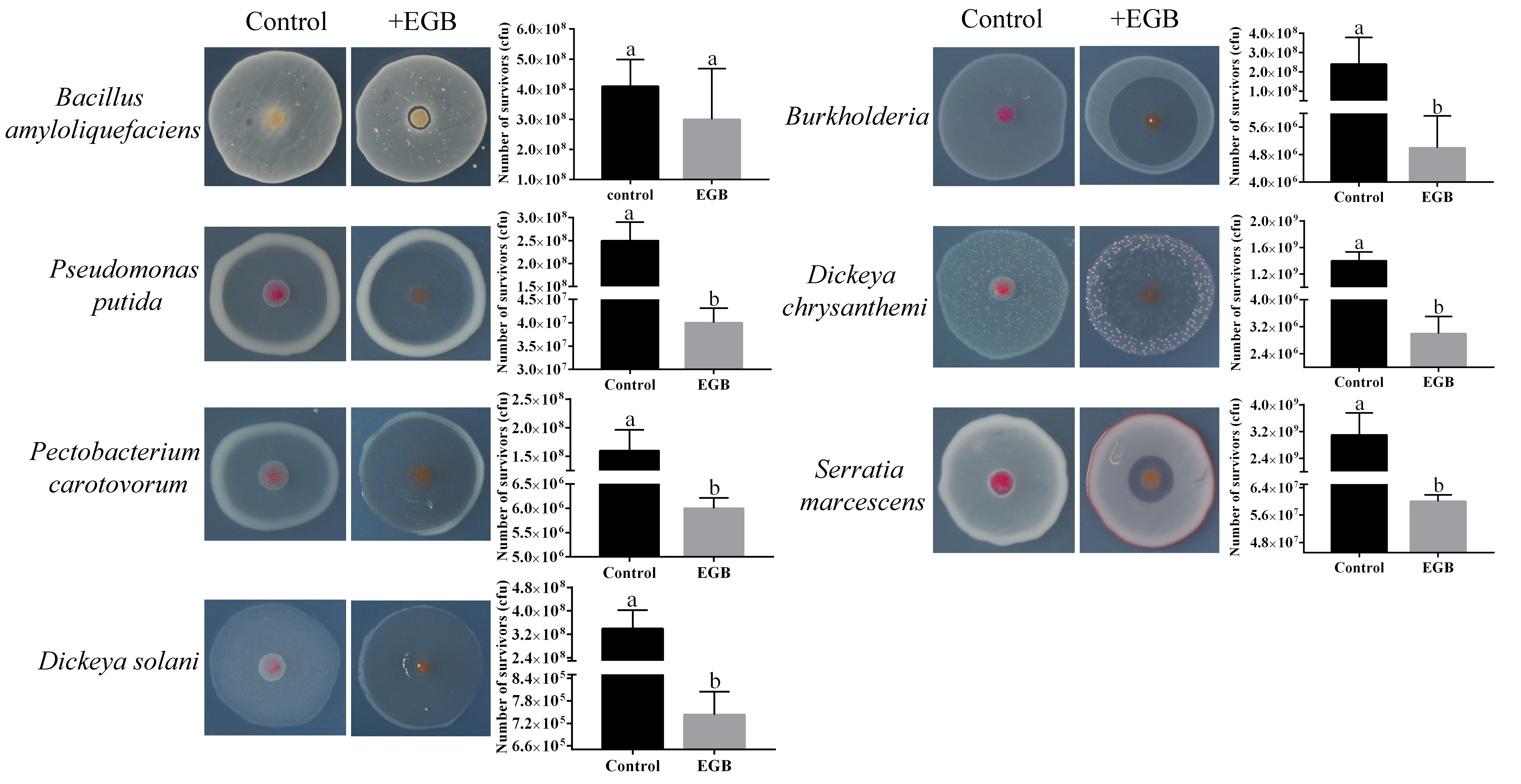

Supplement: Supplementary file 4 — Additional file 3: Figure S2. Predation effect of Corallococcus sp. EGB on bacteria. Strain EGB and other bacteria were cultured in LBS and LB medium, respectively, until reaching an OD600 of 1.0. Then, the EGB and other bacteria were collected by centrifugation at 10 000 × g for 3 min and resuspended in sterile dH2O to 109 CFU/ml. An aliquot comprising 200 μl of each bacterial suspension was dropped onto TPM plates, and 3 μl of the strain EGB suspension was added to the center of the prey colonies. An activated EGB suspension was used as a control. The number of survivors in every colony was assessed by gradient dilution and plate counting after 36 hours of culture on the plate at 30°C. [file 40168_2020_824_MOESM3_ESM.tif]

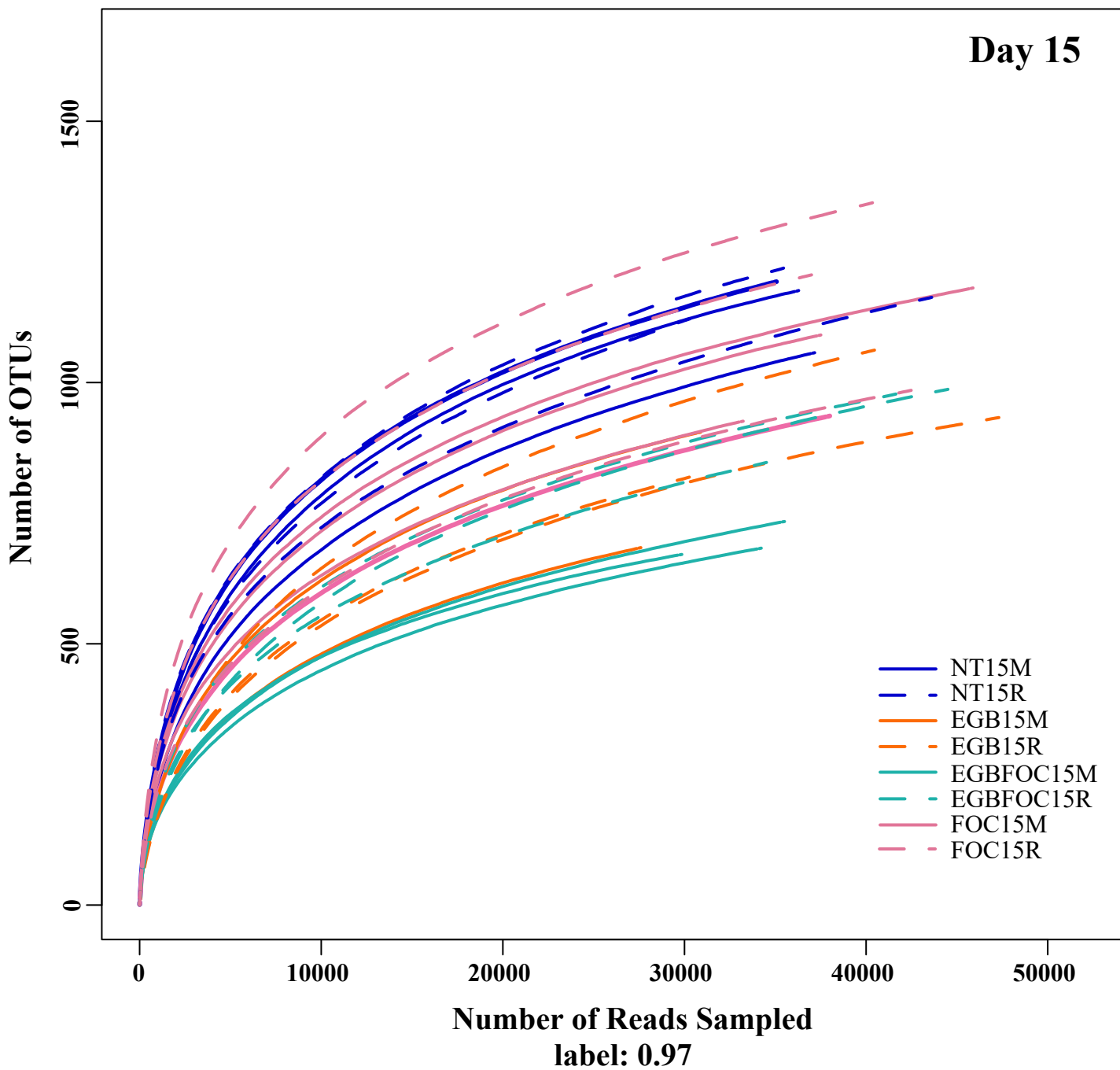

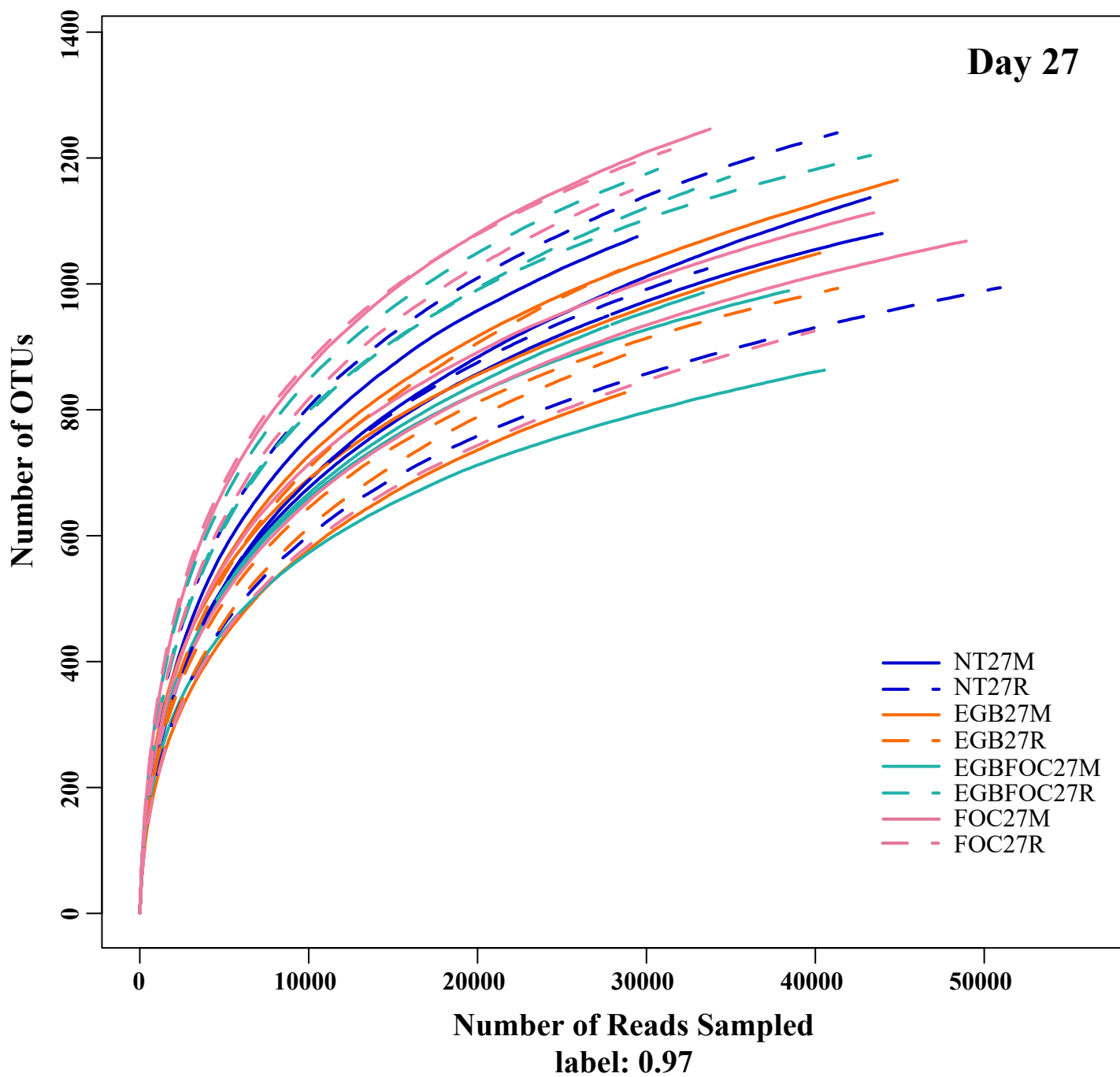

Supplement: Supplementary file 6 — Additional file 5: Figure S3. Rarefaction curves of bacteria depicting the effect on the number of OTUs identified at 97% similarity. A. The soil samples were collected on the 15th day. B. The soil samples were collected on the 27th day. R, the sampling sites surrounding the roots; M, intermediate site between the cucumber root and inoculation site; NT, no FOC or strain EGB solid culture; EGB, strain EGB solid culture only; EGBFOC, both FOC and EGB solid culture; FOC, FOC only. [file 40168_2020_824_MOESM5_ESM.pdf]

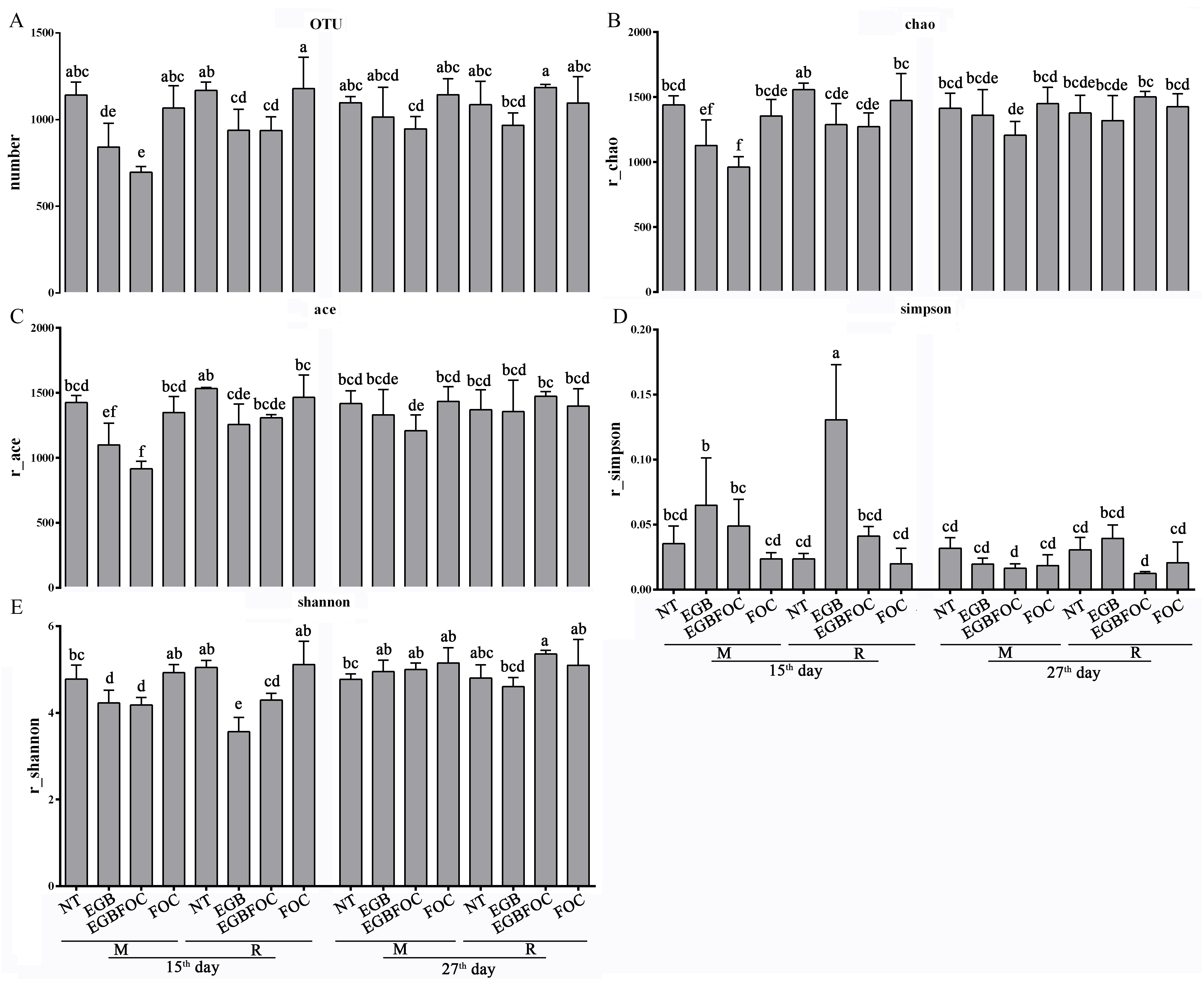

Supplement: Supplementary file 7 — Additional file 6: Figure S4. Comparison of bacterial diversity indices between different treatments. A, B, C, D, and E represent the changes in different diversity indices based on the 16Sv4-v5 gene among different treatments. R, the sampling sites surrounding the roots; M, intermediate site between the cucumber root and the inoculation site; 15, soils sampled on the 15th day; 27, soils sampled on the 27th day; NT, no FOC or strain EGB solid culture; EGB, strain EGB solid culture only; EGBFOC, both FOC and EGB solid culture; FOC, FOC only. Bars indicate the standard deviations of the averages from three replicates. Columns with different letters are significantly different at p ≤ 0.05 according to Duncan’s test. [file 40168_2020_824_MOESM6_ESM.tif]

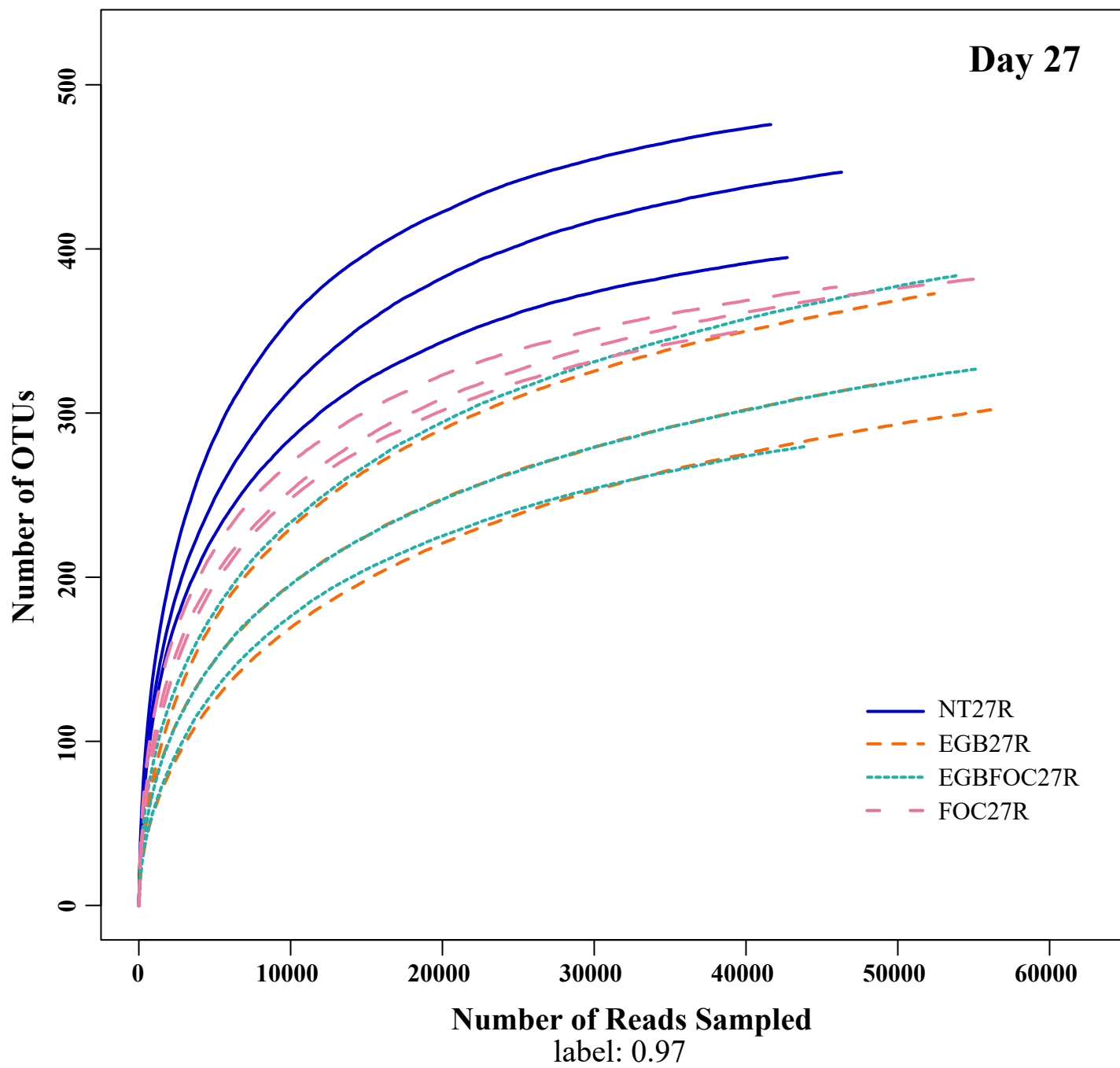

Supplement: Supplementary file 9 — Additional file 8: Figure S5. Rarefaction curves for the number of fungal OTUs with different treatments at 97% similarity. R, the sampling sites surrounding the roots; 27, soils sampled on the 27th day; NT, no FOC or strain EGB solid culture; EGB, strain EGB solid culture only; EGBFOC, both FOC and EGB solid culture; FOC, FOC only. [file 40168_2020_824_MOESM8_ESM.pdf]

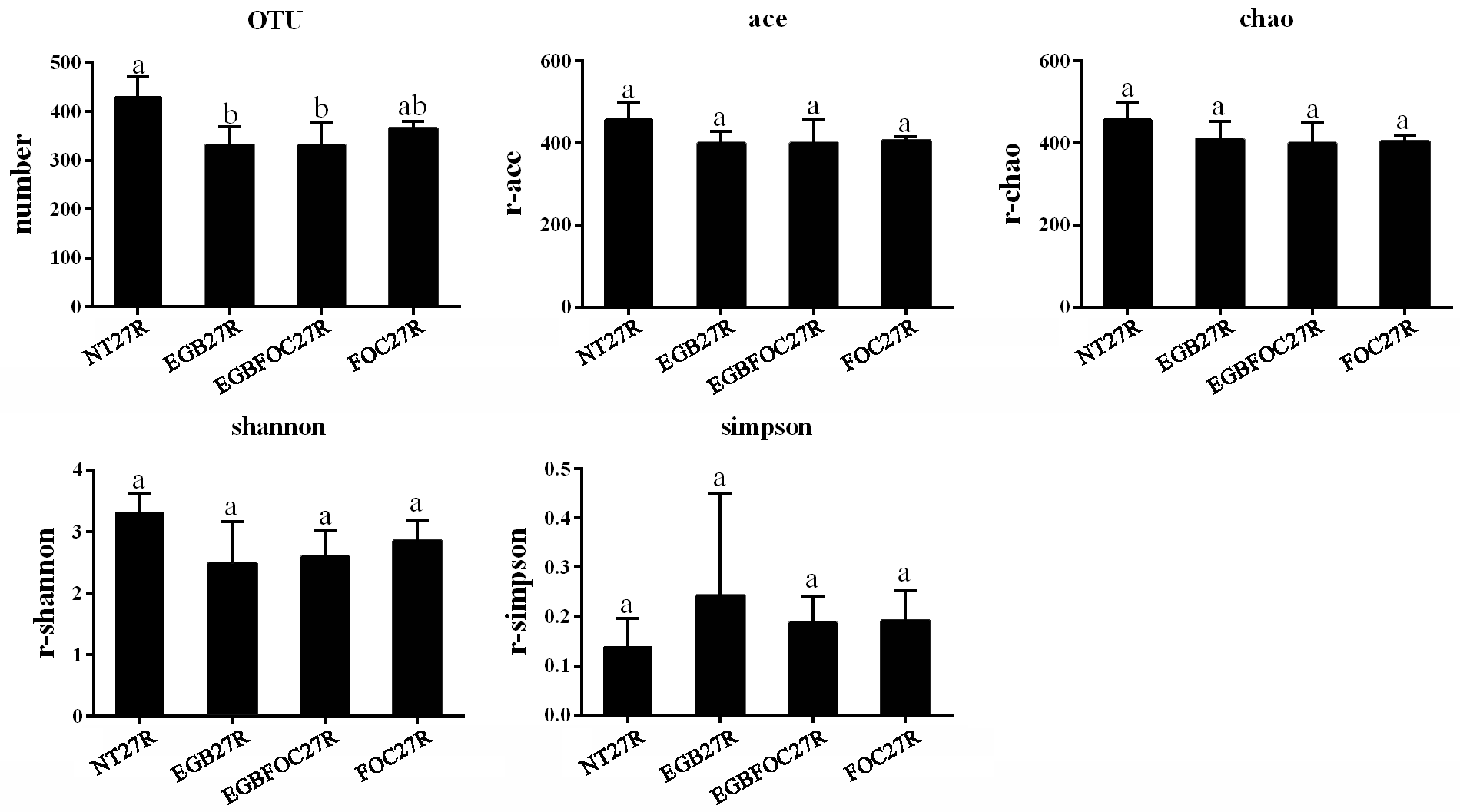

Supplement: Supplementary file 10 — Additional file 9: Figure S6. Comparison of fungal diversity indices between different treatments. R, the sampling sites surrounding the roots; 27, soils sampled on the 27th day; NT, no FOC or strain EGB solid culture; EGB, strain EGB solid culture only; EGBFOC, both FOC and EGB solid culture; FOC, FOC only. Bars indicate the standard deviations of the averages from three replicates. Columns with different letters are significantly different at p ≤ 0.05 according to Duncan’s test. [file 40168_2020_824_MOESM9_ESM.tif]

A

# Treatments

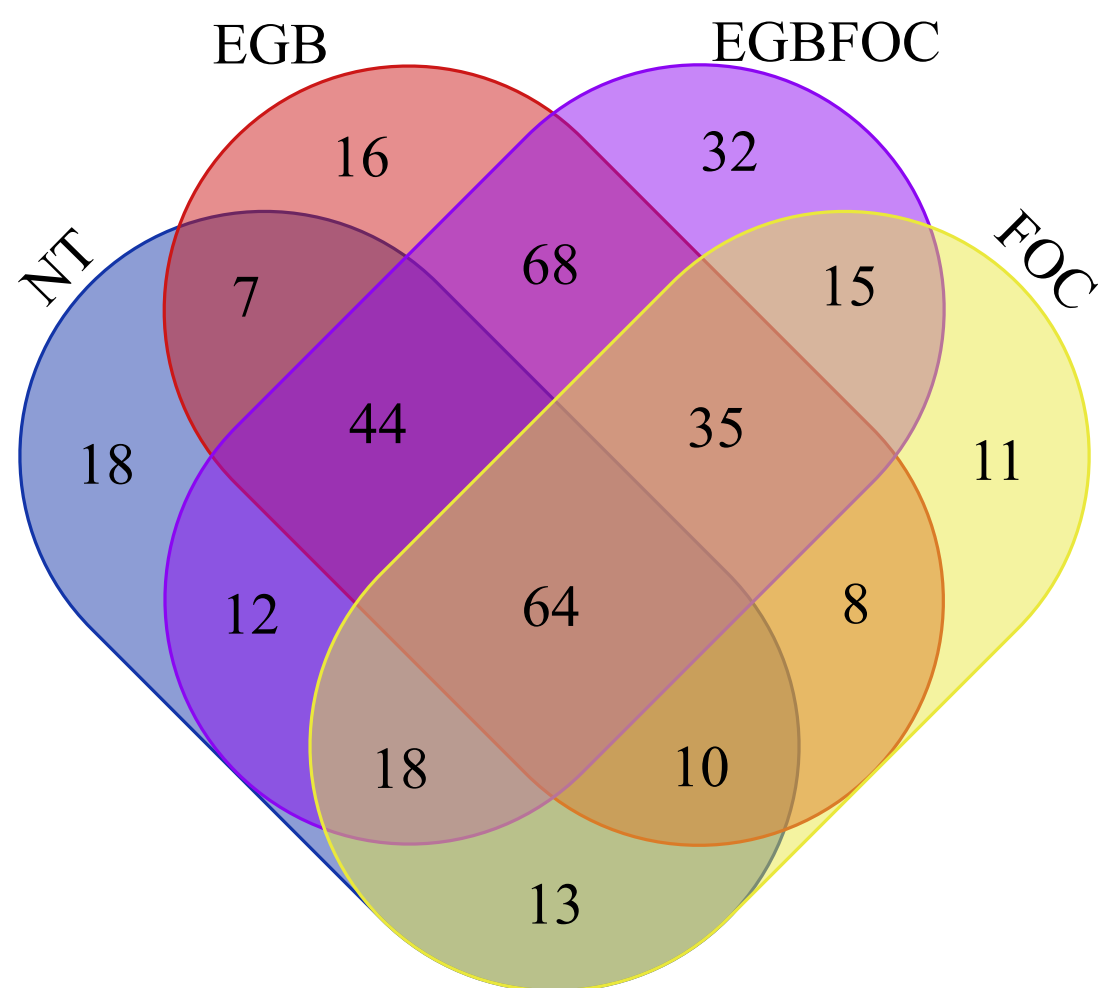

B

# Sampling sites

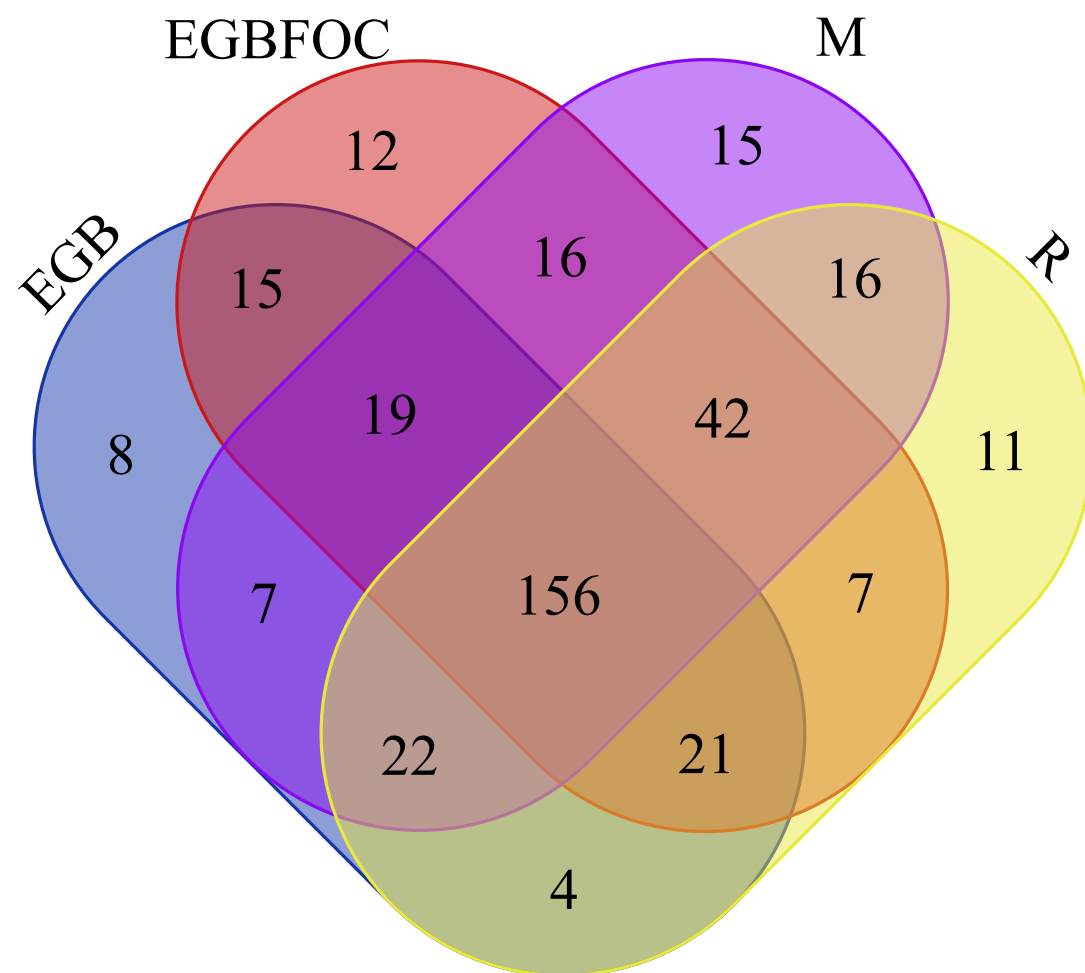

C

# Sampling times

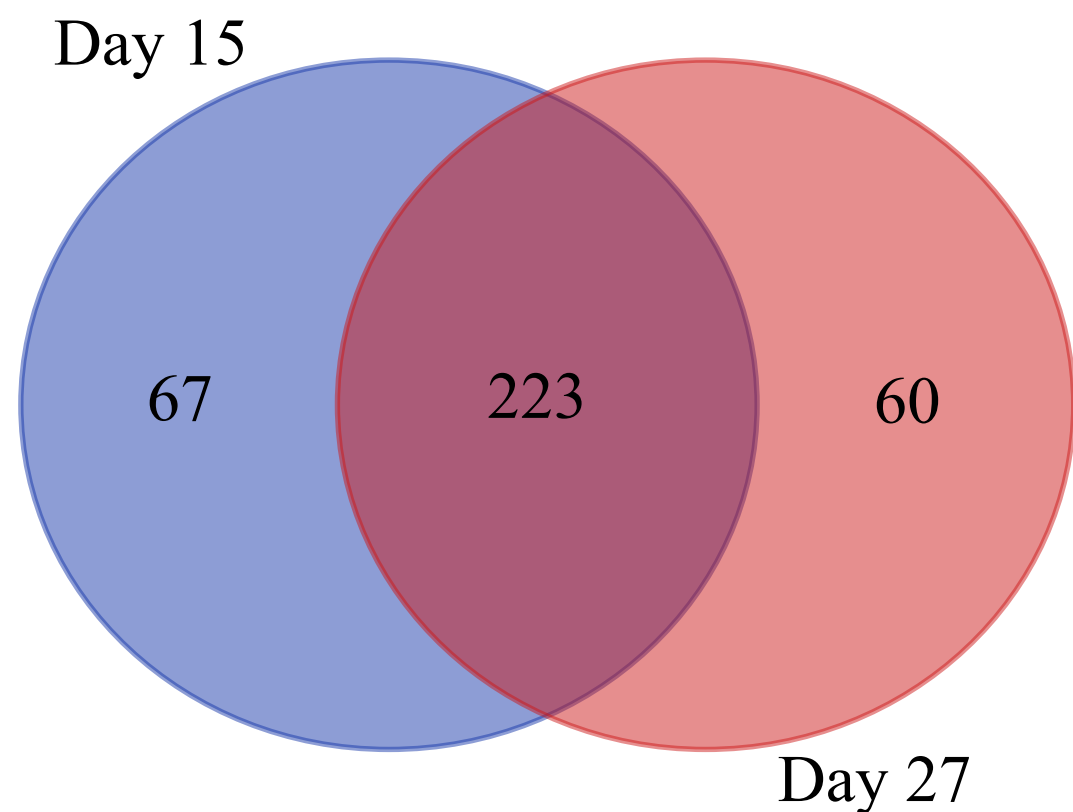

Supplement: Supplementary file 11 — Additional file 10: Figure S7. The common and exclusive bacterial genera in the cooccurrence network (at p < 0.001) of different treatments (A), sampling sites (B) and sampling times (C) are shown by Venn diagrams. NT, no FOC or strain EGB solid culture; EGB, strain EGB solid culture only; EGBFOC, both FOC and EGB solid culture; FOC, FOC only; R, the sampling sites surrounding the roots; M, intermediate site between the cucumber root and the inoculation site; day 15, soils sampled on the 15th day; day 27, soils sampled on the 27th day. [file 40168_2020_824_MOESM10_ESM.pdf]

NT+FOC

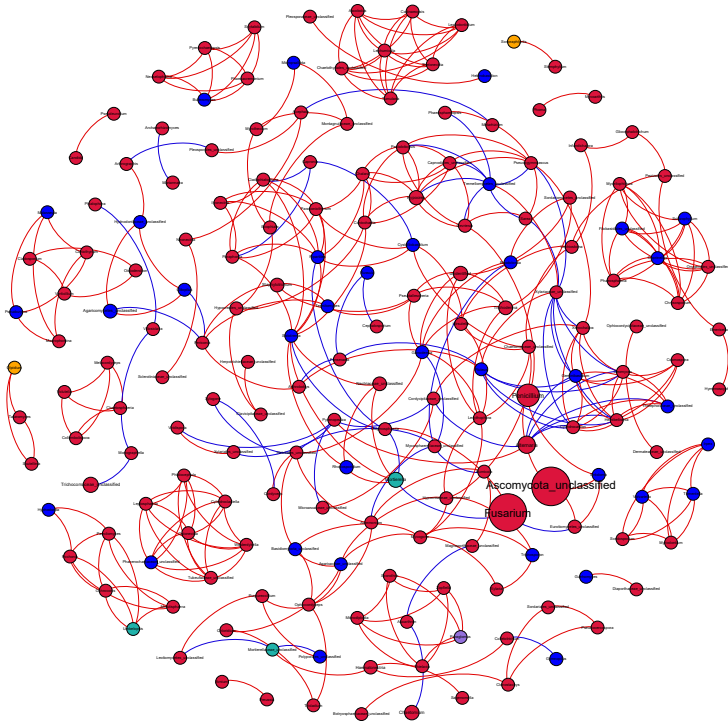

EGB+EGBFOC

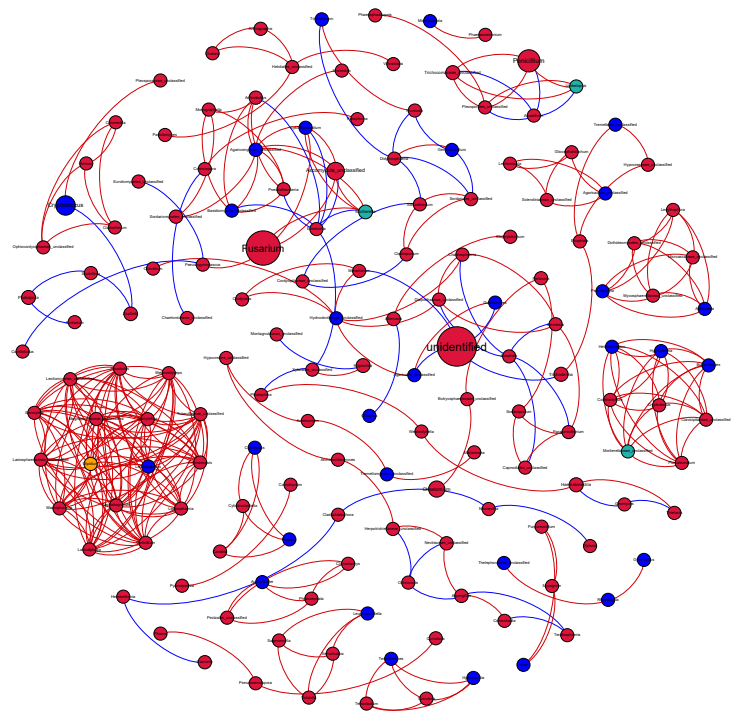

● Ascomycota    ● Basidiomycota    ● Zygomycota  
● Chytridiomycota    ● Glomeromycota

Supplement: Supplementary file 16 — Additional file 15: Figure S8. Network cooccurrence analysis of fungal communities in soil samples with different treatments. To elucidate the effect of strain EGB solid culture on the changes in the soil fungal community, the NT treatment and FOC treatment were classified into group (A) and group (B) comprising the EGB treatment and the EGBFOC treatment. R language (R 2017, 4 version 3.3.3) was run using the R-studio environment, and corr.test() was used for correlation analysis. A connection represents correlations with magnitude>0.7 (positive correlation-red edges) or <− 0.7 (negative correlation-blue edges) that are statistically significant (p < 0.01). Each node represents the taxonomic level of the genus (based on ITS1-ITS2 rRNA), and the size of a node is proportional to the relative abundance of the genus in the sample. Red lines represent a positive correlation, and blue lines represent a negative correlation. Line thickness represents the degree of correlation. [file 40168_2020_824_MOESM15_ESM.pdf]
