# Supplementary material for: A predatory myxobacterium controls cucumber Fusarium wilt by regulating the soil microbial community
Source: Microbiome. 2020 Apr 6;8:49. doi: 10.1186/s40168-020-00824-x (PMC7137222; doi:10.1186/s40168-020-00824-x)
Supplement: Supplementary file 5 — Additional file 4: Table S2. Sample list and sequencing information of the 16SV4–V5 gene libraries. [file 40168_2020_824_MOESM4_ESM.docx]

**Table S2** Sample list and sequencing information of the 16S_V4–V5_ gene libraries.

| Sample | Sequences | Bases(bp) | Average Length(bp) |
| --- | --- | --- | --- |
| NT15M1 | 47134 | 18646544 | 395.61 |
| NT15M2 | 49642 | 19633007 | 395.49 |
| NT15M3 | 42159 | 16677348 | 395.58 |
| EGB15M1 | 44236 | 17491873 | 395.42 |
| EGB15M2 | 46291 | 18336178 | 396.11 |
| EGB15M3 | 41681 | 16501141 | 395.89 |
| EGBFOC15M1 | 43188 | 17059020 | 394.99 |
| EGBFOC15M2 | 51238 | 20254121 | 395.29 |
| EGBFOC15M3 | 46295 | 18323395 | 395.80 |
| FOC15M1 | 55429 | 21934955 | 395.73 |
| FOC15M2 | 51060 | 20205140 | 395.71 |
| FOC15M3 | 44040 | 17425193 | 395.67 |
| NT27M1 | 55575 | 21992715 | 395.73 |
| NT27M2 | 56632 | 22409701 | 395.71 |
| NT27M3 | 40682 | 16096534 | 395.67 |
| EGB27M1 | 57519 | 22748504 | 395.50 |
| EGB27M2 | 43195 | 17075507 | 395.31 |
| EGB27M3 | 56619 | 22401212 | 395.65 |
| EGBFOC27M1 | 59858 | 23660609 | 395.28 |
| EGBFOC27M2 | 56716 | 22438288 | 395.63 |
| EGBFOC27M3 | 49274 | 19464337 | 395.02 |
| FOC27M1 | 47615 | 18844207 | 395.76 |
| FOC27M2 | 43790 | 17336271 | 395.90 |
| FOC27M3 | 56819 | 22488383 | 395.79 |
| NT15R1 | 43309 | 17135624 | 395.66 |
| NT15R2 | 51827 | 20509912 | 395.74 |
| NT15R3 | 48175 | 19063862 | 395.72 |
| EGB15R1 | 59385 | 23483357 | 395.44 |
| EGB15R2 | 50834 | 20105542 | 395.51 |
| EGB15R3 | 41785 | 16520101 | 395.36 |
| EGBFOC15R1 | 59661 | 23612757 | 395.78 |
| EGBFOC15R2 | 40438 | 16008638 | 395.88 |
| EGBFOC15R3 | 59760 | 23644964 | 395.67 |
| FOC15R1 | 50301 | 19914245 | 395.90 |
| FOC15R2 | 51774 | 20483983 | 395.64 |
| FOC15R3 | 46853 | 18545416 | 395.82 |
| NT27R1 | 58966 | 23336668 | 395.76 |
| NT27R2 | 49043 | 19409810 | 395.77 |
| NT27R3 | 58839 | 23281612 | 395.68 |
| EGB27R1 | 44724 | 17683321 | 395.39 |
| EGB27R2 | 44402 | 17566220 | 395.62 |
| EGB27R3 | 51883 | 20512759 | 395.37 |
| EGBFOC27R1 | 58374 | 23080033 | 395.38 |
| EGBFOC27R2 | 54349 | 21502315 | 395.63 |
| EGBFOC27R3 | 43044 | 17035356 | 395.77 |
| FOC27R1 | 52891 | 20929183 | 395.70 |
| FOC27R2 | 41708 | 16508167 | 395.80 |
| FOC27R3 | 47157 | 18671756 | 395.95 |

Note: R, the cucumber roots surrounding site; M, the site between R and inoculation site (I); 15, soil sampled on the 15^th^ day; 27, soil sampled on the 27^th^ day; NT, no FOC or strain EGB solid culture；EGB, strain EGB solid culture only; EGBFOC, both FOC and EGB solid culture；FOC, strain FOC only.
