# Supplementary material for: A predatory myxobacterium controls cucumber Fusarium wilt by regulating the soil microbial community
Source: Microbiome. 2020 Apr 6;8:49. doi: 10.1186/s40168-020-00824-x (PMC7137222; doi:10.1186/s40168-020-00824-x)
Supplement: Supplementary file 8 — Additional file 7: Table S3. Sample list and sequencing information of the 18SITS1-ITS2 gene libraries. [file 40168_2020_824_MOESM7_ESM.docx]

**Table S3** Sample list and sequencing information of the 18S_ITS1-ITS2_ gene libraries.

| Sample | Sequences | Bases(bp) | Average Length(bp) |
| --- | --- | --- | --- |
| NT27R1 | 43064 | 11458539 | 266.08 |
| NT27R2 | 46660 | 12561686 | 269.22 |
| NT27R3 | 42020 | 10869802 | 258.68 |
| EGB27R1 | 52628 | 13790817 | 262.04 |
| EGB27R2 | 56794 | 14901582 | 262.38 |
| EGB27R3 | 49671 | 13165786 | 265.06 |
| EGBFOC27R1 | 44227 | 11275427 | 254.94 |
| EGBFOC27R2 | 55364 | 14190057 | 256.30 |
| EGBFOC27R3 | 54221 | 14165581 | 261.26 |
| FOC27R1 | 55530 | 13759368 | 247.78 |
| FOC27R2 | 40302 | 10297685 | 255.51 |
| FOC27R3 | 46358 | 11919269 | 257.11 |

Note: R, the cucumber roots surrounding site; 27, soil sampled on the 27^th^ day; NT, no FOC or strain EGB solid culture; EGB, strain EGB solid culture only; EGBFOC, both FOC and EGB solid culture；FOC, FOC only.
