# Supplementary material for: A predatory myxobacterium controls cucumber Fusarium wilt by regulating the soil microbial community
Source: Microbiome. 2020 Apr 6;8:49. doi: 10.1186/s40168-020-00824-x (PMC7137222; doi:10.1186/s40168-020-00824-x)
Supplement: Supplementary file 15 — Additional file 14: Table S7. Statistical analysis of the relative abundance of predatory bacteria in different treatment groups. Note: NT, no FOC or strain EGB solid culture, EGB, strain EGB solid culture only; EGBFOC, both FOC and EGB solid culture; FOC, FOC only. Data were expressed as the means ± standard deviations in each treatment. In the same row, values followed by the same letters are not significantly different at p ≤0.05 according to Duncan’s test. [file 40168_2020_824_MOESM14_ESM.docx]

**Table S7** Statistical analysis of the relative abundance of predatory bacteria in different treatment groups

|  | NT | EGB | EGBFOC | FOC | F | p-value |
| --- | --- | --- | --- | --- | --- | --- |
| *Sorangium* | 0.001186±0.000814^a^ | 0.000281±0.000142^b^ | 0.000820±0.00119^ab^ | 0.00148±0.000823^a^ | 4.635 | 0.007 |
| *Haliangium* | 0.000719±0.000533^a^ | 0.000602±0.000393^a^ | 0.000838±0.000705^a^ | 0.000741±0.000767^a^ | 0.296 | 0.828 |
| *Fibrisoma* | 0.0±0.0^b^ | 0.0000187±0.0000302^b^ | 0.000111±0.000191^a^ | 0.00000216±0.00000748^b^ | 3.572 | 0.021 |
| *Cytophaga* | 0.000137±0.000278^a^ | 0.00000633±0.000111^a^ | 0.00000680±0.0000169^a^ | 0.00000216±0.00000748^a^ | 1.779 | 0.165 |
| *Corallococcus* | 0.0000217±0.0000244^b^ | 0.000761±0.000938^a^ | 0.000507±0.000861^ab^ | 0.0000389±0.0000686^b^ | 3.908 | 0.015 |
| *Nannocystis* | 0.0±0.0^b^ | 0.000305±0.00053^a^ | 0.000444±0.000415^a^ | 0.0±0.0^b^ | 5.296 | 0.003 |
| *Flavobacterium* | 0.0000282±0.0000323^b^ | 0.0200±0.0163^a^ | 0.0152±0.0298^a^ | 0.000253±0.000786^b^ | 4.380 | 0.009 |
| *Stenotrophomonas* | 0.0000369±0.0000342^b^ | 0.0953±0.0887^a^ | 0.0367±0.0510^b^ | 0.0000605±0.0000628^b^ | 9.279 | 0.000 |
| *Cupriavidus* | 0.0000608±0.0000849^a^ | 0.00574±0.00431^b^ | 0.00402±0.00475^b^ | 0.000136±0.000267^a^ | 9.435 | 0.000 |
| *Myxococcales_unclassified* | 0.0000673±0.0000623^ab^ | 0.0000211±0.0000320^b^ | 0.0000159±0.0000245^b^ | 0.0000950±0.00012^a^ | 3.492 | 0.023 |
| *Byssovorax* | 0.0000760±0.0000797^a^ | 0.0000562±0.0000611^a^ | 0.0000612±0.000114^a^ | 0.0000410±0.0000475^a^ | 0.396 | 0.757 |
| *Sandaracinus* | 0.0±0.0^b^ | 0.0000258±0.0000406^a^ | 0.0000204±0.0000369^ab^ | 0.00000216±0.00000748^ab^ | 2.616 | 0.063 |
| *Streptomycetaceae_unclassified* | 0.000354±0.000193^a^ | 0.000314±0.000231^a^ | 0.000288±0.000198^a^ | 0.000250±0.000147^a^ | 0.603 | 0.616 |
| *Bdellovibrio* | 0.00000217±0.00000752^a^ | 0.0±0.0^a^ | 0.0000136±0.0000338^a^ | 0.00000215±0.00000748^a^ | 1.451 | 0.241 |
| *lysobacter* | 0.00000434±0.0000101^b^ | 0.0176±0.0256^a^ | 0.000449±0.000444^b^ | 0.00000216±0.00000748^b^ | 5.568 | 0.003. |
| *Microvirga* | 0.00000652±0.0000162^b^ | 0.00212±0.00153^a^ | 0.00156±0.00140^a^ | 0.00000432±0.0000101^b^ | 13.066 | 0.000 |

**Note:** NT, no FOC or strain EGB solid culture; EGB, strain EGB solid culture only; EGBFOC, both FOC and EGB solid culture；FOC, FOC only. Data were expressed as the means ± standard deviations in each treatment. In the same row, values followed by the same letters are not significantly different at *P* ≤ 0.05 according to Duncan’s test. The genus represented by the red text indicated a significant increase in the relative abundance of the genus after the application of EGB solid fermentation.
