# Supplementary material for: A predatory myxobacterium controls cucumber Fusarium wilt by regulating the soil microbial community
Source: Microbiome. 2020 Apr 6;8:49. doi: 10.1186/s40168-020-00824-x (PMC7137222; doi:10.1186/s40168-020-00824-x)
Supplement: Supplementary file 17 — Additional file 16: Table S8. Bacterial prey species used in the predation experiments. Note: Strains were maintained in our laboratory at -80°C with 15% glycerol (Sigma, Japan) as a cryoprotectant. [file 40168_2020_824_MOESM16_ESM.docx]

**Table S****8** Bacterial prey species used in the predation experiments.

| Bacteria preys |
| --- |
| *Bacillus amyloliquefaciens* |
| *Pseudomonas putida* |
| *Pectobacterium carotovorum* |
| *Dickeya solani* |
| *Burkholderia* |
| *Dickeya chrysanthemi* |
| *Serratia marcesecens* |

Note: Strains were maintained in our laboratory at -80℃ with 15% glycerol (Sigma, Japan) as a cryoprotectant.
