# Supplementary material for: A predatory myxobacterium controls cucumber Fusarium wilt by regulating the soil microbial community
Source: Microbiome. 2020 Apr 6;8:49. doi: 10.1186/s40168-020-00824-x (PMC7137222; doi:10.1186/s40168-020-00824-x)
Supplement: Supplementary file 18 — Additional file 17: Table S9. Corresponding sample names in the manuscript and in NCBI depository. [file 40168_2020_824_MOESM17_ESM.docx]

**Table S9** Corresponding sample names in the manuscript and in NCBI depository

| Bacteria | | Fungi | |
| --- | --- | --- | --- |
| Samples’ names in NCBI | Samples’ names in text | Samples’ names in NCBI | Samples’ names in text |
| M15B1 | NT15M1 | R27b1 | NT27R1 |
| M15B2 | NT15M2 | R27b2 | NT27R2 |
| M15B3 | NT15M3 | R27b3 | NT27R3 |
| M15C1 | EGB15M1 | R27c1 | EGB27R1 |
| M15C2 | EGB15M2 | R27c2 | EGB27R2 |
| M15C3 | EGB15M3 | R27c3 | EGB27R3 |
| M15D1 | EGBFOC15M1 | R27d1 | EGBFOC27R1 |
| M15D2 | EGBFOC15M2 | R27d2 | EGBFOC27R2 |
| M15D3 | EGBFOC15M3 | R27d3 | EGBFOC27R3 |
| M15E1 | FOC15M1 | R27e1 | FOC27R1 |
| M15E2 | FOC15M2 | R27e2 | FOC27R2 |
| M15E3 | FOC15M3 | R27e3 | FOC27R3 |
| M27B1 | NT27M1 |  |  |
| M27B2 | NT27M2 |  |  |
| M27B3 | NT27M3 |  |  |
| M27C1 | EGB27M1 |  |  |
| M27C2 | EGB27M2 |  |  |
| M27C3 | EGB27M3 |  |  |
| M27D1 | EGBFOC27M1 |  |  |
| M27D2 | EGBFOC27M2 |  |  |
| M27D3 | EGBFOC27M3 |  |  |
| M27E1 | FOC27M1 |  |  |
| M27E2 | FOC27M2 |  |  |
| M27E3 | FOC27M3 |  |  |
| R15b1 | NT15R1 |  |  |
| R15b2 | NT15R2 |  |  |
| R15b3 | NT15R3 |  |  |
| R15c1 | EGB15R1 |  |  |
| R15c2 | EGB15R2 |  |  |
| R15c3 | EGB15R3 |  |  |
| R15d1 | EGBFOC15R1 |  |  |
| R15d2 | EGBFOC15R2 |  |  |
| R15d3 | EGBFOC15R3 |  |  |
| R15e1 | FOC15R1 |  |  |
| R15e2 | FOC15R2 |  |  |
| R15e3 | FOC15R3 |  |  |
| R27b1 | NT27R1 |  |  |
| R27b2 | NT27R2 |  |  |
| R27b3 | NT27R3 |  |  |
| R27c1 | EGB27R1 |  |  |
| R27c2 | EGB27R2 |  |  |
| R27c3 | EGB27R3 |  |  |
| R27d1 | EGBFOC27R1 |  |  |
| R27d2 | EGBFOC27R2 |  |  |
| R27d3 | EGBFOC27R3 |  |  |
| R27e1 | FOC27R1 |  |  |
| R27e2 | FOC27R2 |  |  |
| R27e3 | FOC27R3 |  |  |

Note: In order to facilitate the better understanding of the experimental design and to annotate the legends more clearly, we replace the original samples’ names with the new ones in the manuscript. R, the sampling sites surrounding the roots; M, intermediate site between the cucumber root and the inoculation site; 15, soil sampled on the 15^th^ day; 27, soil sampled on the 27^th^ day; NT, no FOC or strain EGB solid culture; EGB, strain EGB solid culture only; EGBFOC, both FOC and EGB solid culture；FOC, FOC only.
