# Supplementary material for: A predatory myxobacterium controls cucumber Fusarium wilt by regulating the soil microbial community
Source: Microbiome. 2020 Apr 6;8:49. doi: 10.1186/s40168-020-00824-x (PMC7137222; doi:10.1186/s40168-020-00824-x)
Supplement: Supplementary file 19 — Additional file 18: Image of Figure 6 with information of other microorganisms. [file 40168_2020_824_MOESM19_ESM.pdf]

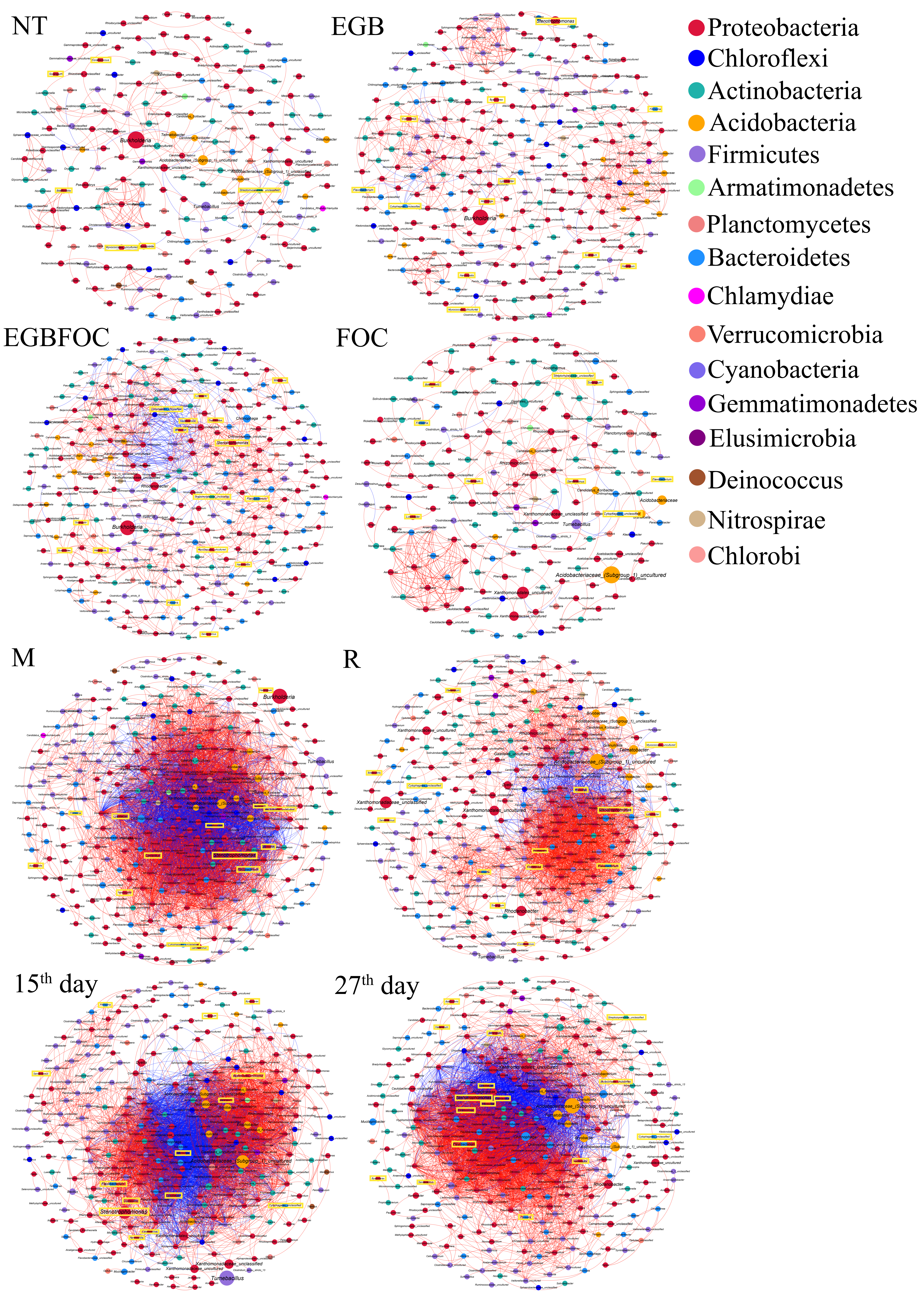
NT
EGB
EGBFOC
FOC
M
R
15th day
27th day
Proteobacteria
Chloroflexi
Actinobacteria
Acidobacteria
Firmicutes
Armatimonadetes
Planctomycetes
Bacteroidetes
Chlamydiae
Verrucomicrobia
Cyanobacteria
Gemmatimonadetes
Elusimicrobia
Deinococcus
Nitrospirae
Chlorobi
